# Spatially Explicit Correction of Simulated Urban Air Temperatures Using Crowdsourced Data

**DOI:** 10.1175/JAMC-D-22-0142.1

**Published:** 2023-11

**Authors:** Oscar Brousse, Charles Simpson, Owain Kenway, Alberto Martilli, E. Scott Krayenhoff, Andrea Zonato, Clare Heaviside

**Affiliations:** a Institute of Environmental Design and Engineering, University College London, London, United Kingdom; b Centre for Advanced Research Computing, University College London, London, United Kingdom; c Center for Energy, Environment and Technology (CIEMAT), Madrid, Spain; d School of Environmental Sciences, University of Guelph, Guelph, Ontario, Canada; e Department of Civil, Environmental and Mechanical Engineering, University of Trento, Trento, Italy

**Keywords:** Heat islands, Bias, Mesoscale models, Model evaluation/performance, Urban meteorology, Machine learning

## Abstract

Urban climate model evaluation often remains limited by a lack of trusted urban weather observations. The increasing density of personal weather sensors (PWSs) make them a potential rich source of data for urban climate studies that address the lack of representative urban weather observations. In our study, we demonstrate that carefully quality-checked PWS data not only improve urban climate models’ evaluation but can also serve for bias correcting their output prior to any urban climate impact studies. After simulating near-surface air temperatures over London and south-east England during the hot summer of 2018 with the Weather Research and Forecasting (WRF) Model and its building Effect parameterization with the building energy model (BEP–BEM) activated, we evaluated the modeled temperatures against 402 urban PWSs and showcased a heterogeneous spatial distribution of the model’s cool bias that was not captured using official weather stations only. This finding indicated a need for spatially explicit urban bias corrections of air temperatures, which we performed using an innovative method using machine learning to predict the models’ biases in each urban grid cell. This bias-correction technique is the first to consider that modeled urban temperatures follow a nonlinear spatially heterogeneous bias that is decorrelated from urban fraction. Our results showed that the bias correction was beneficial to bias correct daily minimum, daily mean, and daily maximum temperatures in the cities. We recommend that urban climate modelers further investigate the use of quality-checked PWSs for model evaluation and derive a framework for bias correction of urban climate simulations that can serve urban climate impact studies.

## 1 Introduction

Although the decades following the 1960s have seen an increase in the body of literature on urban climates (Oke et al. 2017), the scales of applicability and the transferability of the studies’ outcomes are often limited. This can partially be attributed to the lack of observations representative of the variety of existing urban climates in cities. To address this limitation, two major solutions were proposed over the past 20 years: first, the development of urban surface energy balance coupled to regional climate models (e.g., Masson 2000; Martilli et al. 2002; Wouters et al. 2016), and second, the increased interest toward crowdsourced and low-cost weather sensors (e.g., Muller et al. 2015; Chapman et al. 2017; Fenner et al. 2017; Meier et al. 2017). After proper validation and parameterization, urban climate models (UCMs) offer an unprecedented opportunity to represent the impact of cities on a wide variety of weather variables at very high spatial and temporal resolutions. This has been further supported by the recent development of global standardized land-use/land-cover datasets designed for urban climate studies that permit their parameterization in cities formerly deprived of these data [see the World Urban Dataset and Access Portal Tool (WUDAPT) project; Ching et al. (2018), Demuzere et al. (2022)]. Likewise, after proper filtering and quality control (Napoly et al. 2018; Fenner et al. 2021), crowdsourced personal weather sensors (PWSs) permit the extension of sensing networks into urban environments that were formerly not studied despite the fact that PWSs often do not meet the standards imposed by official meteorological offices for implementation of weather stations. Several studies have demonstrated their range of applications since then (e.g., Fenner et al. 2019; Venter et al. 2020; Potgieter et al. 2021; Benjamin et al. 2021; Varentsov et al. 2021; Venter et al. 2021; Brousse et al. 2022).

One of the major limitations induced by the lack of official weather stations in cities is that quantifying existing uncertainties as a function of urban climate archetype is not feasible. This means that urban environments are poorly evaluated and have a higher chance of being inaccurately modeled because studies currently assume that UCMs will perform similarly for all types of urban environments that compose a city. In face of this challenge, crowdsourced PWSs could improve the evaluation of UCMs, as Hammerberg et al. (2018) demonstrated over Vienna. But the potential of PWSs may even be greater, particularly when used jointly with or in parallel to UCMs. In fact, a recent study by Sgoff et al. (2022) improved the weather forecasting of the Icosahedral Nonhydrostatic (ICON) model (Zängl et al. 2015) at a horizontal resolution of 2 km over Germany by assimilating the data provided by PWSs for air temperature and relative humidity at 2-m height. Although data assimilation occurs at runtime, PWSs could also be used to bias correct urban climate simulations as a postprocessing step. Oleson et al. (2018) already noted the need for a global dataset of urban weather observations to properly bias correct simulated urban climates. We indeed expect urban climate simulations to have systematic biases that can be induced for a variety of reasons, such as urban canopy parameters (Demuzere et al. 2017; Hammerberg et al. 2018; Zonato et al. 2020), complexity of urban climate models (Grimmond et al. 2011; Loridan and Grimmond 2012; Lipson et al. 2021), time at which the simulation is initialized (Bassett et al. 2020), choice of initial and boundary conditions for lateral and vertical forcing (Brisson et al. 2016), or choice of model parameterizations—such as the two evaluated in this work (see section 2). Hence, the UCM will always present a certain degree of uncertainty that has to be allowed for prior to performing urban climate impact studies that use climatic variables derived from modeled simulations to estimate the impact of the urban climate on other things (e.g., mortality and biodiversity). Using PWSs could thus be beneficial for obtaining realistic urban weather data of present and future urban climates that can be used to perform urban climate impact studies and guide decision-making.

In this study, we propose to leverage the increasingly dense network of PWSs over southeast England since 2015 (Brousse et al. 2022) to evaluate and bias correct urban climate simulations that were run for the hot summer of 2018—the hottest summer on average in the United Kingdom. Common practices in bias correction include adding the mean bias to the modeled variable distribution or applying a separate correction to each quantile of the distribution (Maraun and Widmann 2018). Model biases are usually measured at official weather stations at rural sites, thereby assuming that the urban heat island phenomenon is accurately represented by the UCM (e.g., Lauwaet et al. 2015; Oleson et al. 2018). Some studies, however, tried considering the urban effect by linearly transforming the bias-correction coefficient via an urbanization ratio calculated at each grid cell, as in Wouters et al. (2017) over Belgium. Assuming that urban climate simulations biases cannot be linearly related to the urban fraction only [here defined as the total nonnatural fraction of a model grid that composes an urban canyon (street, roofs, building walls)], we decided to test whether urban in situ observations can be used to perform an urban-specific bias correction of air temperatures driven by machine learning.

We chose to use machine learning regressors to correct the air temperature biases because machine learning allows us to perform spatially explicit bias corrections that are directly derived from the observed biases at all PWS locations and that are related to a set of spatially explicit covariates. Machine learning regressors of ranging complexities allow for the statistical discretization of a single relationship between the covariates and the variety of biases. To our knowledge, such a technique has never been proposed as a viable approach for bias correction of urban climate simulations, probably because of the lack of observations in urban areas. We hereby hypothesize that such an innovative bias-correction method would be beneficial for urban heat impact studies by improving the UCM outputs on which they rely. Such innovations are needed to better assess the heat burden in cities (Nazarian et al. 2022).

To respond to these issues through the scope of urban near-surface temperatures, we (i) evaluated the ability of the complex three-dimensional UCM embedded in the Weather Research and Forecasting (WRF) Model—the building effect parameterization coupled with its building energy model (BEP–BEM)—to accurately represent the urban impact on air temperatures under two boundary layer schemes for the summer of 2018 in southeast England using official weather stations and PWSs separately to show their added value for detecting spatially heterogeneous urban temperature biases; (ii) used machine learning regressions to predict the models’ daily air temperature biases in the urban environment and bias correct the two simulations suggested in step i—which allowed us to determine an optimal time step at which the bias correction should be performed to optimize the outputs; and (iii) compared the two bias-corrected products against the predicted daily air temperatures using only PWS measurements to investigate how realistic the bias-corrected products are. In parallel, to illustrate the benefit gained from the bias correction for impact studies, we showcase how the bias correction leads to different population weighted temperatures in the Greater London area. We also estimated the number of PWSs that are necessary to achieve optimal machine learning regressors’ performance and tested the added value of official weather stations for bias correction.

It is important to consider that our study does not try to estimate how a bias-corrected modeled product is better compared to a predicted product from observations for urban climate impact studies. We hereby simply try to demonstrate that any urban climate impact work that is based on urban climate modeling should pursue a spatially explicit bias correction specific to urban areas.

## 2 Methods

### a Model setup and region of interest

**Fig. 1:**
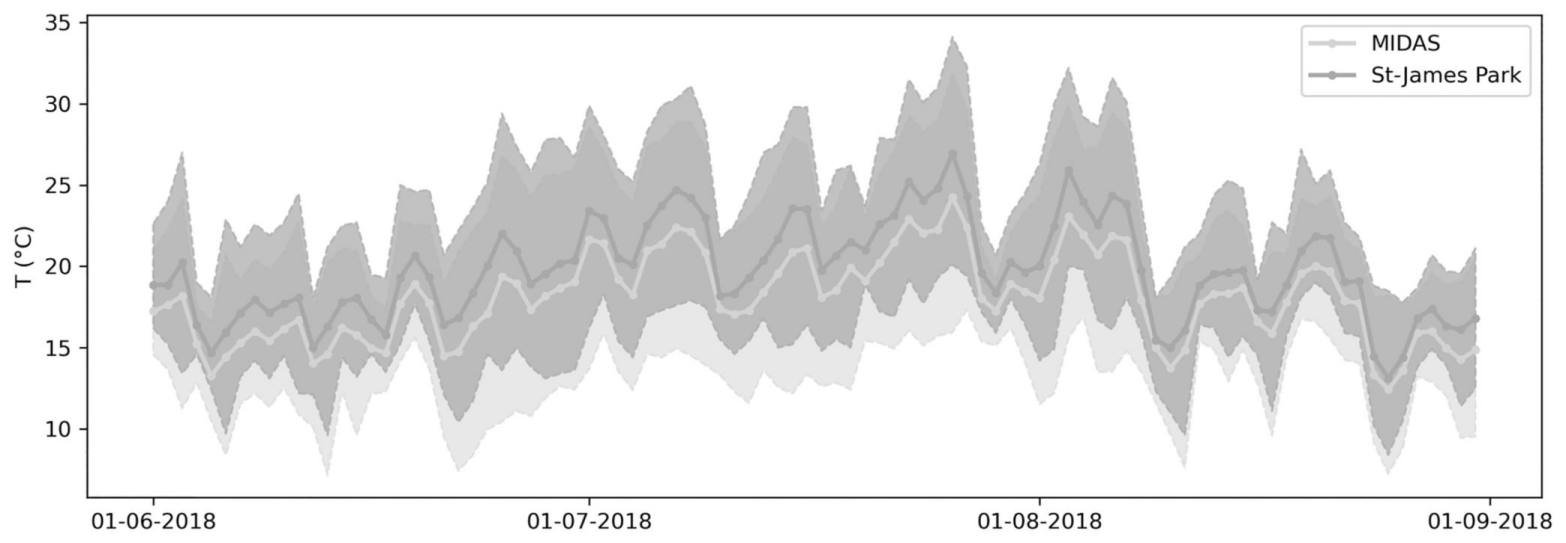
Diurnal ranges of temperatures observed by the Met Office MIDAS AWSs. The urban St. James’ Park station in central London (dark gray) is always hotter than the average temperature of all MIDAS stations in southeast England (light gray) for daily average, minimum, and maximum temperatures. The thick lines represent the daily average temperature, and the shading represents the spread between daily maxima and minima.

We focused our study on the southeastern parts of England, centered over the metropolis of London, host to approximately 9 million inhabitants. We chose to model the impact of urbanization on 2-m air temperature in London during the summer of 2018, since it was the hottest summer on average in the United Kingdom (McCarthy et al. 2019). During the British Isles heatwaves, maximum daily temperatures often surpassed 30°C (Fig. 1) with a maximum of 34.4°C measured at London’s Heathrow Airport on 26 July. This former record has yet been broken in 2019 and 2022.

**Fig. 2:**
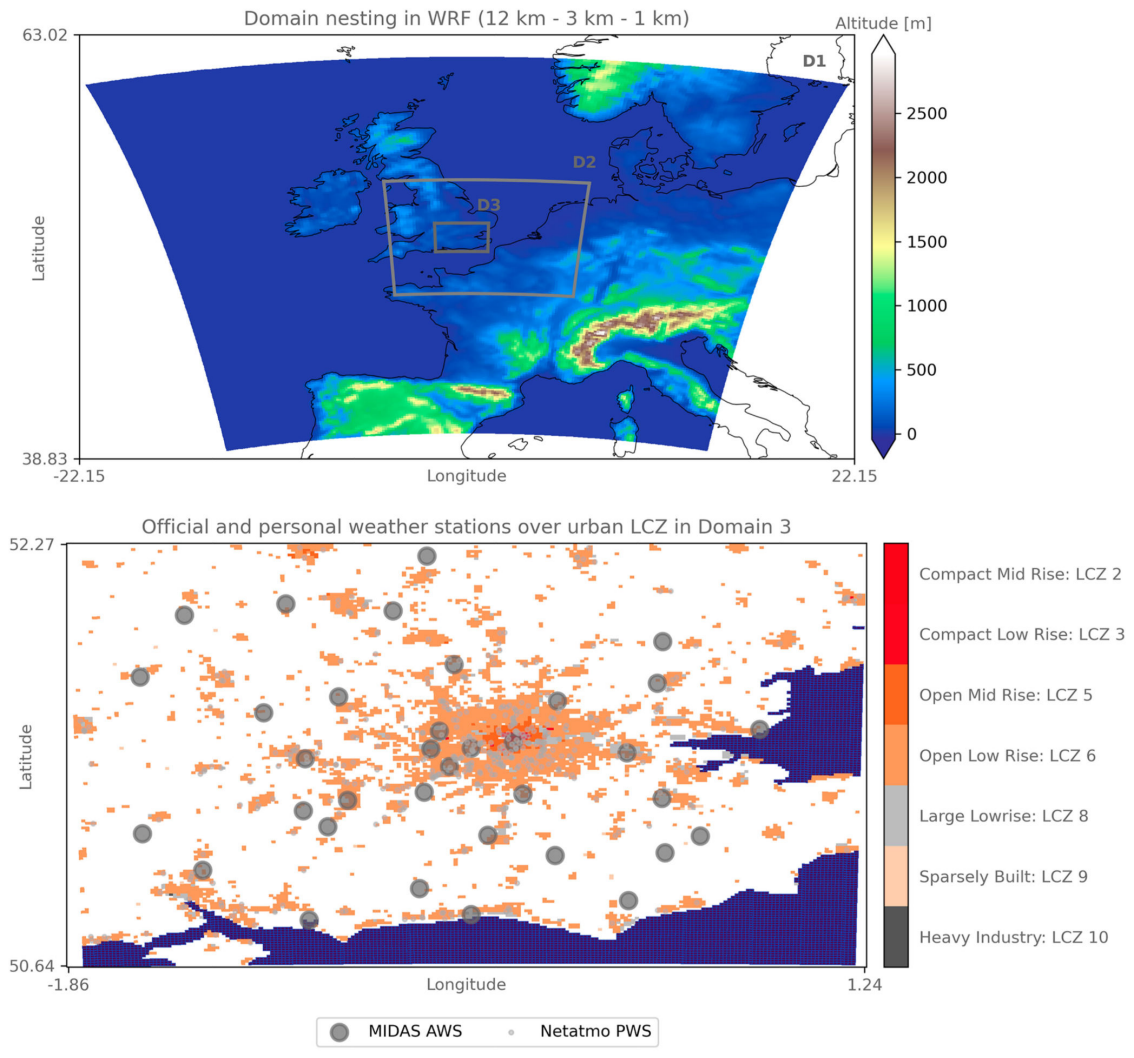
(top) Domain nesting and (bottom) urban land cover in the inner domain. The WRF nesting strategy consists of three nested domains at 12-km (D1), 3-km (D2), and 1-km (D3) horizontal resolution. The altitude is plotted to highlight the flat terrain of southeast England covered in D3. In the lower panel, the resulting urban landcover in D3 after using the WUDAPT-TO-WRF Python tool is presented in the form of LCZ. The MIDAS official AWSs and the Netatmo PWSs used for the evaluation of the model and the subsequent bias correction using PWSs only are overlayed in gray. The sea is shown in blue in the bottom panel, and coastlines are drawn in black in the top panel.

To model the impact of the urban areas of London and southeast England on local meteorology, we used the WRF regional climate model, version 4.3, and activated the embedded BEP (Martilli et al. 2002) urban climate model with its partner BEM (Salamanca et al. 2010; Salamanca and Martilli 2010)—hereinafter referred to as BEP–BEM. We ran the model at a horizontal resolution of 1 km 3 × km following a two-way nesting strategy where the outer domain is forced by ERA5 6-hourly data at 25 km with 199 × 199 grid points and the two intermediate domains are run at horizontal resolutions of 9 and 3 km with 252 × 241 and 210 × 180 grid points, respectively (Fig. 2, top). Initial land surface conditions were provided by the default MODIS 5-arc-s land-use dataset provided by the WRF community, whereas sea surface temperatures were updated 6-hourly out of ERA5. No lake models were activated, hence meaning that inland freshwater bodies are given the MODIS Water land cover class and are not updated on 6-hourly time steps as sea surface temperatures. We ran the model in parallel over 200 CPUs using restarts every 4 days of simulation. We started the simulations on 25 May 2018 and ended them on 31 August 2018, considering the first 7 days of simulation as spinup time.

All domains used the same physical and dynamical parameterizations we obtained from preliminary testing done over the two hottest days of the summer 2018—26 and 27 July 2018 (see appendix A). We thereby used the WRF single-moment 3-class microphysics scheme (Hong et al. 2004), the Dudhia shortwave and RRTM longwave schemes (Dudhia 1989; Mlawer et al. 1997), and the revised MM5 surface layer scheme (Jiménez et al. 2012). In the first domain, the Kain–Fritsch convection scheme was activated (Kain 2004) and then turned off in the second and third domains, which were at convection-permitting scales. We set the model top at 50 hPa with an additional 5000-m damping layer and subdivided the atmosphere into 56 vertical layers. We used the Noah-MP land surface scheme (Niu et al. 2011; Yang et al. 2011) in its default parameterization over four soil layers.

Urban canopy parameters required by the WRF BEP–BEM Model were provided via the newly standardized WUDAPT-TO-WRF (W2W) Python package developed by Demuzere et al. (2021), following the FORTRAN version used by Brousse et al. (2016). This allowed the transfer of spatially explicit morphological urban canopy parameters suitable for urban climate simulations via local climate zone (LCZ) maps covering the inner domain (Fig. 2, bottom). We use the European LCZ map by Demuzere et al. (2019). Thermal and radiative parameters are also directly derived from the LCZ classification and follow those used by Stewart et al. (2014), who used these parameters for the city of Basel, Switzerland. Each parameter for roofs, walls, and roads is related to each modal LCZ of the 1-km grid cell via the URB-PARM_LCZ.TBL (see Table 1). We decided to keep the roughness length for momentum and the lower boundary for temperatures of roofs, walls, and roads identical across each LCZ. We fixed the roughness length at 1.00 × 10^−4^ m for walls and at 0.01 m for roofs and roads, respectively. This does not mean that the effective roughness length at the bulk level does not differ between urban morphologies. Although materials composing them are considered identical in the drag they impose on the flow, their density and height will matter. Urban canyons with buildings above 25 m and another with buildings below 5 m will effectively have a different roughness length. For the boundary temperatures, we set it at 299 K for the roofs and the walls, respectively, and at 293 K for the road. We chose to deactivate the air conditioning in our simulation because air-conditioning systems are not common in residential areas across London and surrounding cities, which compose the majority of the land use/land cover.

In this study, two potential planetary boundary layer (PBL) schemes are compared in terms of performance and need of bias correction: the commonly used Bougeault–Lacarrère scheme (BouLac; Bougeault and Lacarrere 1989) for urban simulations that use BEP–BEM and the recently coupled YSU scheme to BEP–BEM (Hong et al. 2006; Hong and Kim 2008; Hendricks et al. 2020). Although we found that the latter performed better over the two hottest days of summer 2018 (see appendix A), we decided to keep a simulation with BouLac as YSU has only been applied over Dallas (Wang and Hu 2021), whereas BouLac has been used in multiple studies already (e.g., Salamanca et al. 2011, 2012; Gutiérrez et al. 2015; Tewari et al. 2017; Mughal et al. 2019). The Mellor–Yamada–Janjić (MYJ; Janjić 1994, 2001) scheme, also available for BEP–BEM simulations, is disregarded in this study since this PBL scheme is especially used for mountainous terrain (Zonato et al. 2022), and we are modeling the relatively flat terrain of southeast England.

### b Model evaluation prior to bias correction

We evaluated the model’s performances against 35 official weather stations’ measurements of air temperature at 2 m Thermal conductivity obtained from the Met Office Integrated Data Archive System (MIDAS) network (Sunter 2021; UKMO 2021; Fig. 1, lower panel). To address the issue of lack of official observations among the urban environment, we used Netatmo PWSs to complement the model evaluation (Fig. 1, lower panel). The Netatmo PWS measurements were obtained through the Netatmo developer application programming interface (API) and were collected for all PWSs contained within the innermost domain of WRF and that were running over the 2015–20 period [more information can be found in Brousse et al. (2022)]. Prior to the evaluation, unrealistic PWS measurements were filtered out using the Crowd-QC v1.0 R package from Grassmann et al. (2018). This statistical quality check and filtering method is based on the assumption that the whole set of PWSs should be regarded as a reference to individual stations’ specificities. Through four main obligatory quality checks—potentially complemented by three optional checks—erroneous data are removed. Details of this filtering method can be found in other publications like Napoly et al. (2018) and/or Brousse et al. (2022) who used the same dataset over London. For the summer 2018, the filtering reduced the dataset from 935 potential PWSs to 909 potential stations over the whole domain. Such filtering has already been applied over several studies, including a large-scale study by Venter et al. (2021) over a European city, and has recently been ameliorated into the CrowdQC1 package (Fenner et al. 2021). The purpose of this study is not to test the effect of the PWS quality check on the model evaluation and bias correction.

After quality checking the PWSs, we also added an additional filtering where we removed PWSs that did not have sufficient temporal data coverage and that were not located in an urban pixel according to WRF. Only PWSs that have less than 4 h per day without data and that are located in urban pixels with an urban fraction greater than 0 are retained—where the WRF land use/land cover at 1-km horizontal resolution refers to an LCZ. This ensures that we do not include measurements that are not representative of the daily variations in air temperatures or built-up environments. Additionally, the prior filtering performed using the CrowdQC package also ensures that measurements that are not representative of outdoor thermal variations (e.g., indoor sensors) or that are resulting from defective sensors are taken out. Overall, the filtering step is necessary to ensure that our model outputs are evaluated against measurements of sufficient quality and that the subsequent bias correction is deprived of unnecessary noise in the data that could lower its performance. This resulted in a sample of 402 PWSs usable for model evaluation and bias correction. Out of these, 354 were located in WRF grids classified as LCZ 6, 30 in LCZ 5, 8 in LCZ 2, 6 in LCZ 8, 3 in LCZ 9, and 1 in LCZ 3.

Each model simulation was evaluated using a set of common statistical indicators: the root-mean-square error (RMSE), the mean absolute error (MAE), the mean bias (MB) error, Spearman’s coefficient of correlation (*r*), and the square of Pearson’s coefficient of correlation (*r*^2^). These metrics are obtained using the Python scikit-learn and scipy’s stats packages from Pedregosa et al. (2011) and Virtanen et al. (2020).

### c Bias correction using personal Netatmo weather stations

In our study, we propose an innovative method to bias correct urban temperatures at a horizontal scale of 1 km by using machine learning regression. The advantage of using machine learning regression compared to more common bias-correction strategies (e.g., the definition of a single bias coefficient) is that we are able to relate our model output biases out of spatially varying and explicit sets of parameters. In our case, we make the assumption that the spatial variation in the bias of the model is dependent only upon the spatial morphological inputs to the UCM. These include the urban fraction, the surface height, the average building height, the building surface to plan area fraction (*λb*), the plan area fraction (*λp*), and the frontal area fraction (*λf*). Using this set of predictive covariates, we train our regressors to predict the bias in the modeled air temperature at 2 m (T2) based on observed biases at urban PWS locations. In this way, we are able to bias correct the modeled temperatures in each urban pixel based on the predicted bias (T2 – bias_pred_). Our bias correction does not make use of official MIDAS weather stations as their use is considered detrimental to the bias correction following an analysis on sample size and sensor types given in appendix B.

We chose to bias correct the simulated daily minimum, maximum, and average T2 (T2_min_, T2_max_, and T2_mean_, respectively) using filtered PWS observations in London and southeast England. Daily temporal scale is considered optimal as it combines a higher spatial density of measurements compared to hourly data and a lower computational requirement; it is also a commonly used temporal scale for urban heat impact studies. Daily minimum and maximum air temperatures at 2 m are defined following the Met Office Hadley Centre definition: minimum temperature observed from 0900 local time (LT) of the previous day *d* – 1 to 0900 LT of the *d* day and maximum temperature observed from 0900 LT of the *d* day to 0900 LT of the next day *d* + 1 (Hollis et al. 2019).

We test the ability of six different regressors of increasing complexity available in the Python scikit-learn packages (Pedregosa et al. 2011) to predict the model bias based on WRF spatial urban canopy parameters only. These regressors are dummy regression (which simply returns the mean bias), linear regression, Ridge regression, Lasso regression, random forest regression, and gradient boosting regression. Each of the different regressors, except the dummy regression, offers a set of parameters that can be fine-tuned to increase each regressor’s performance. Hence, prior to running the daily bias correction, we use a 5-*K*-fold cross validation using the Grid Search CV package from scikit-learn in Python to evaluate the impact of hyperparameter tuning on the regressors’ performances based on RMSE, MAE, and *r*^2^. The cross validation is done over the summertime average daily mean temperature bias from the YSU run only, for computational reasons. We retain RMSE as the refitting score to better capture the spatial spread and extremes of T2. The resulting parameterizations are given in Table 2. We chose to keep the same hyperparameter tuning for all bias correction and predictions to ease comparability between the outcomes.

Once the hyperparameter tuning is done and prior to performing the final bias correction, we test if the bias correction is beneficial for palliating to the models’ bias and if it also benefits from training the regressors at the daily time step or if a training using the time-mean bias is sufficient. To perform this evaluation using the same metrics as in the model evaluation, we bootstrap each regressor 25 times per day, randomly sampling 80% of the PWS locations that had data available on that day as training and keeping the remaining 20% as testing—for both the daily minimum, daily maximum, and daily average and their respective summertime-mean average. We then first average all bootstrapped T2_BC at the testing PWS sites before performing a subsequent averaging to obtain an average T2_BC at the daily time step representative of all randomly selected testing PWS sites. These are evaluated against the daily average of all observed temperature at the PWS sites—for daily minimum, maximum, and average. In short, we are measuring how well the two different types of bias correction perform under all regressors for capturing the daily variation (*n* = 92 days) of temperature on average.

After this final step, we bias correct both the BouLac and YSU runs using 100% of the measured biases and related covariates at PWS locations to compare the spatial outcomes. We also predict T2 out of PWSs’ observed T2 with the same set of covariates used to predict the model bias to illustrate how divergent each bias-corrected model output is to a simplified predicted T2 that is not a derivative of any model constraint. Because more refined and complex techniques exist to predict air temperature from PWSs and very high-resolution Earth observations (e.g., Venter et al. 2020, 2021), we do not evaluate these predicted temperatures that should simply be considered as an illustration of how bias-corrected products are similar or divergent to observational data.

Last, to illustrate the potential benefit of modeled air temperature bias correction prior to urban heat impact studies, we calculate the average population weighted temperatures—based on the United Kingdom census data from 2011—in Greater London before and after the bias correction.

## 3 Results

### a WRF simulation evaluation

When we evaluate the two model simulations against MIDAS official weather stations only, they perform similarly, demonstrating a systematic negative bias of ~0.55°C on average (Table 3). The average correlation with the automatic weather stations (AWSs) following the squared Pearson’s *r*^2^ is of 0.77 for BouLac and 0.79 for YSU, whereas using Spearman’s *r*, it is of 0.86 and 0.88, respectively. A slight decreased performance is found in urban pixels for YSU, with an average MAE of 1.83°C and a negative MB of 0.79°C compared to BouLac’s 1.82°C for MAE and −0.56°C for MB. In general, the bias is more important at night, and in nonurban stations, performances are similar. Hence, looking only at the models’ performances using standard in situ observations does not provide information on which model represents the urban climate more accurately.

**Fig. 3:**
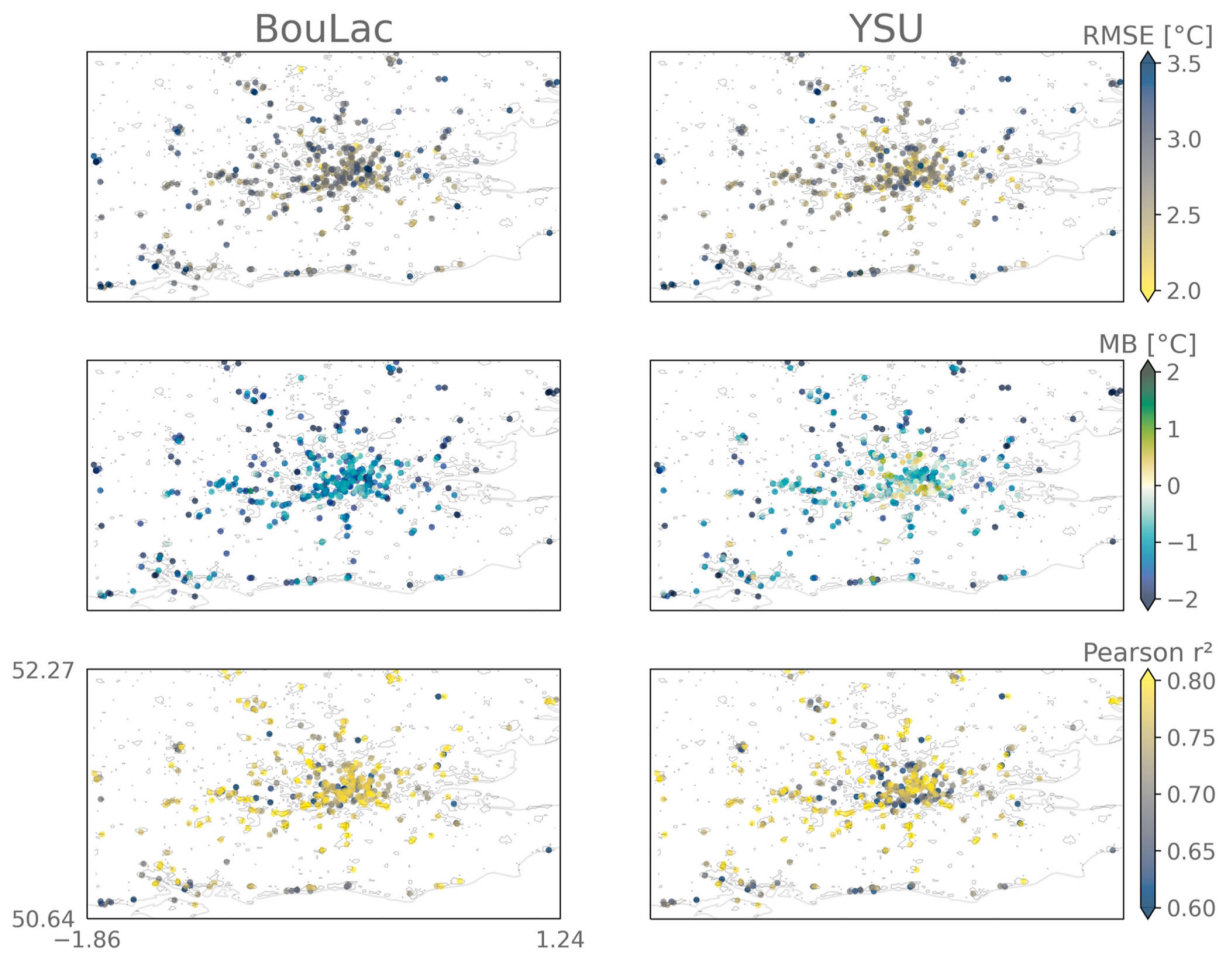
Performance metrics calculated at the location of each citizen PWS for the two model simulations using different planetary boundary layer schemes (YSU and BouLac). The metrics are calculated over the whole summer 2018 with hourly outputs of near-surface air temperature at 2 m. RMSE and MB are given in degrees Celsius (°C). The coefficients of correlation measured with the squared Pearson’s *r* are also provided. MAE and Spearman’s *r* are given in Fig. C1 in appendix C to increase clarity.

On the other hand, comparison with PWS observations identifies differences in performance in urban areas between the models, as shown by the performance metrics plotted in Figs. 3a and C1. The BouLac simulation has a stronger cool bias of −1.46° ± 0.6°C on average in the urban area, compared to YSU’s MB of −0.97° ± 0.81°C. RMSE and MAE are similar, with values of 2.79° ± 0.36°C and 2.19° ± 0.31°C for BouLac and 2.65° ± 0.40°C and 2.14° ± 0.34°C for YSU. These metrics are consistent with the MIDAS observations, highlighting a systematic cool bias of the model and a coefficient of determination (*r*^2^) of 80%. Importantly, the variability in the model’s performance is greater in the YSU run—reflected by greater standard deviations of performance metrics—and, in the BouLac simulation, the metrics are more heterogeneously distributed among the urban area. Indeed, when we look at the YSU simulation, we can see that the model has a smaller MB in suburban areas and a greater MB in the city center. Yet, in parallel, the correlation with the PWSs is lower in the suburban areas and higher in the center of the city. This could mean that YSU accurately represents the urban temperatures on average due to compensating effects, which we do not intend to evaluate in this study. Nevertheless, this shows how PWSs are beneficial for capturing the spatial heterogeneity of each model’s performance and therefore supports the use of spatially varying bias correction.

### b Bias correction of urban climate simulations

Over our domain of study covering southeast England during the summer 2018, both models are subject to a cold negative bias of ~−0.5°C on average according to official stations and from ~−1.0° to ~−1.5°C according to PWSs. But as demonstrated above, the bias of the models against PWS observations has substantial spatial variation, and so, the bias correction for urban heat impact studies should be spatially explicit.

**Fig. 4:**
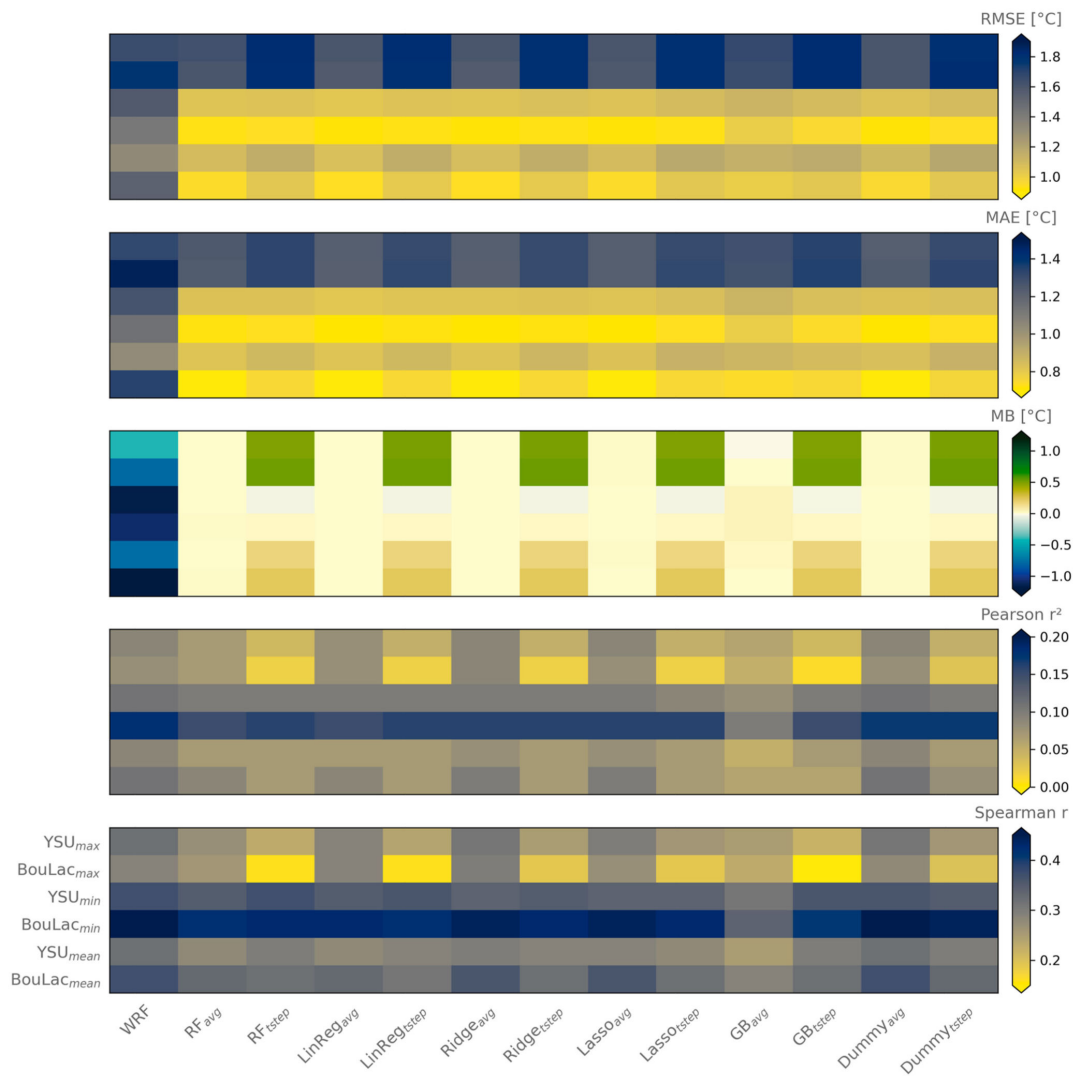
Performance metrics for the model prior to the bias correction (WRF) and all the different regressions (random forest: RF; linear regression: LinReg; Ridge regression: Ridge; Lasso regression: Lasso; gradient boosting: GB; and dummy regression: Dummy). The different regressions are assigned a suffix: “avg” for regressions that were trained on the summertime-mean average of daily minimum, daily mean, or daily maximum temperatures and “tstep” for those that were trained with the temperatures at each daily time step.

We find that each machine learning regressor gives a similar performance (Fig. 4; values numerically given in Tables C1 and C2 in appendix C). All bias corrections were, however, beneficial compared to the original outputs from the WRF Model, reducing RMSE, MAE, and MB by 0.29°, 0.32°, and 1.02°C on average. The bias correction was most efficient for daily minimum temperatures and less for daily maximum temperatures, where RMSE was not diminished—if not slightly increased (by 0.05°C for YSU daily maximum temperatures, for example)—by the time-step bias correction. Interestingly, the spatial correlation between the bias-corrected and observed temperatures are low, with values ranging from around 0.02 to 0.2 for the squared Pearson’s *r* and from around 0.15 to 0.45 for Spearman’s *r*. This can be expected as machine learning algorithms have difficulties representing a time-varying variable with static spatial elements only (Georganos et al. 2021; Venter et al. 2021). Unexpectedly, we find that the training at the daily time step does not outperform the training at the summertime mean in terms of spatial correlation with the heat distribution across London. Nonetheless, if we take the average daily minimum, daily mean, and daily maximum temperatures of all PWSs and compare it to the modeled temperatures, we find that the time-step bias correction is closer to the observations (Figs. C2–C4). Last, we find that greater model performance is achieved with a minimum of ~24% (96 PWSs) of the whole sample of PWSs and that official weather stations are detrimental to the regressors’ performance (see appendix B).

**Fig. 5:**
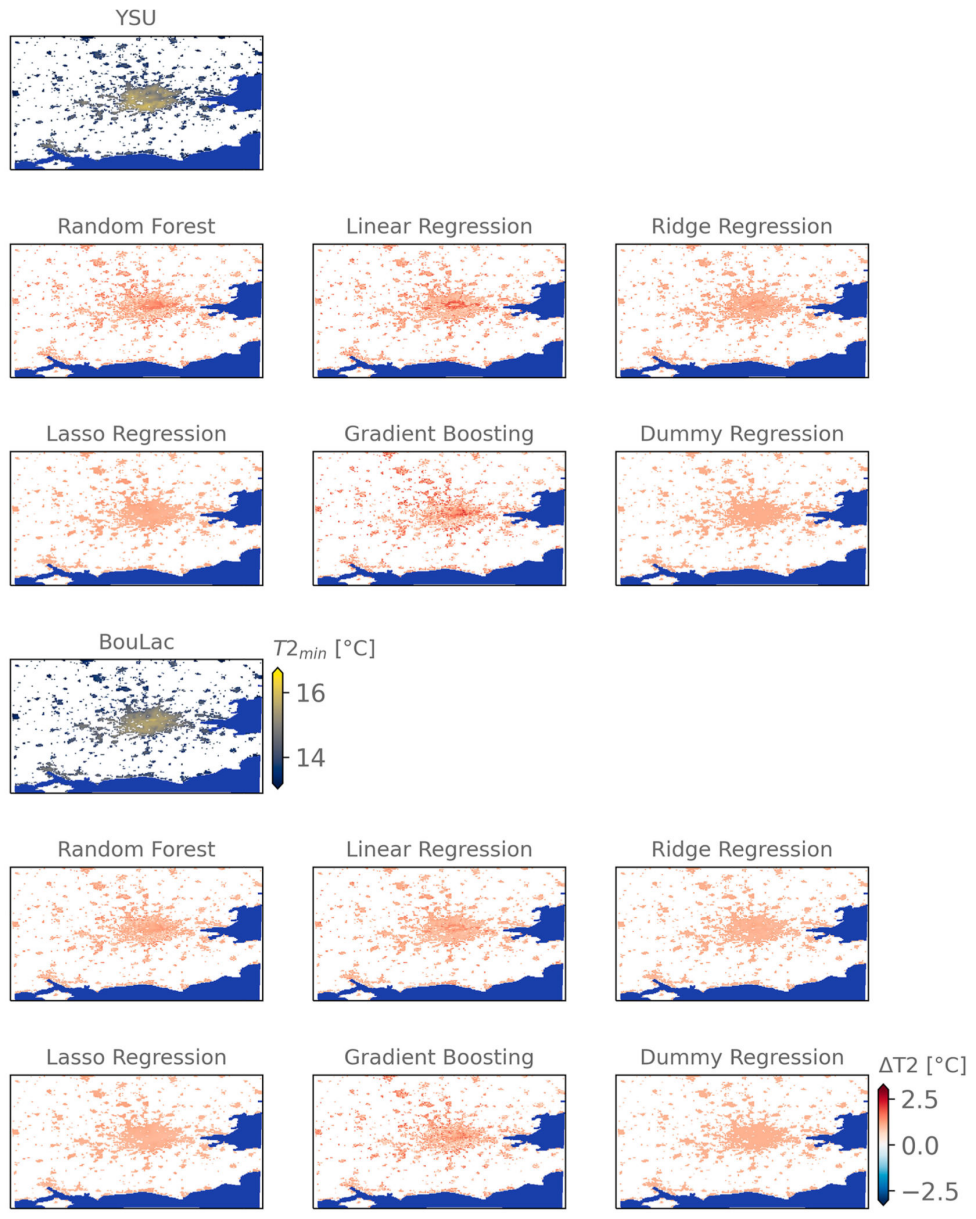
All regressions propose different bias corrections (ΔT2) of the average modeled absolute daily minimum urban temperature (T2_min_). Differences of bias correction are observed between the runs with different planetary boundary layer schemes (BouLac and YSU). The center of London is subject to a stronger bias correction. Rural lands are masked in gray, and the seas are shown in blue. Bias corrections of daily mean and maximum temperatures are given in Figs. C5 and C6.

Comparing the spatial differences of the bias-corrected products related to the complexities of each regressor, we find that although each regressor is performing similarly on average, important disparities are found between the outputs. For example, when looking at the average bias correction imposed to daily minimum temperatures after training the regressors at each time step, the Lasso and Ridge regressors impose a flat bias correction, similar to the dummy regression, whereas the random forest and gradient boosting regressors’ degrees of freedom result in a spatially diverse bias correction (Figs. 5, C5, and C6). Besides, the linear regression imposes an average bias correction spatially correlated to the modal LCZ. In general, the signal is consistent across each regressor, apart from the Lasso regression and the dummy regression, where, for YSU, central London requires a stronger bias correction by 1°–2°C compared to the suburban areas where the bias correction is around 0.5°C; for BouLac, the central bias correction is lower than YSU. We find that these spatial tendencies are also found for daily maximum and daily average temperatures, defending our hypothesis of a systematic bias correlated to spatially explicit input parameters. The spatial differences in bias correction are, however, less important for daily maximum temperatures, which is the time at which the urban heat island is also expected to be the lowest.

**Fig. 6:**
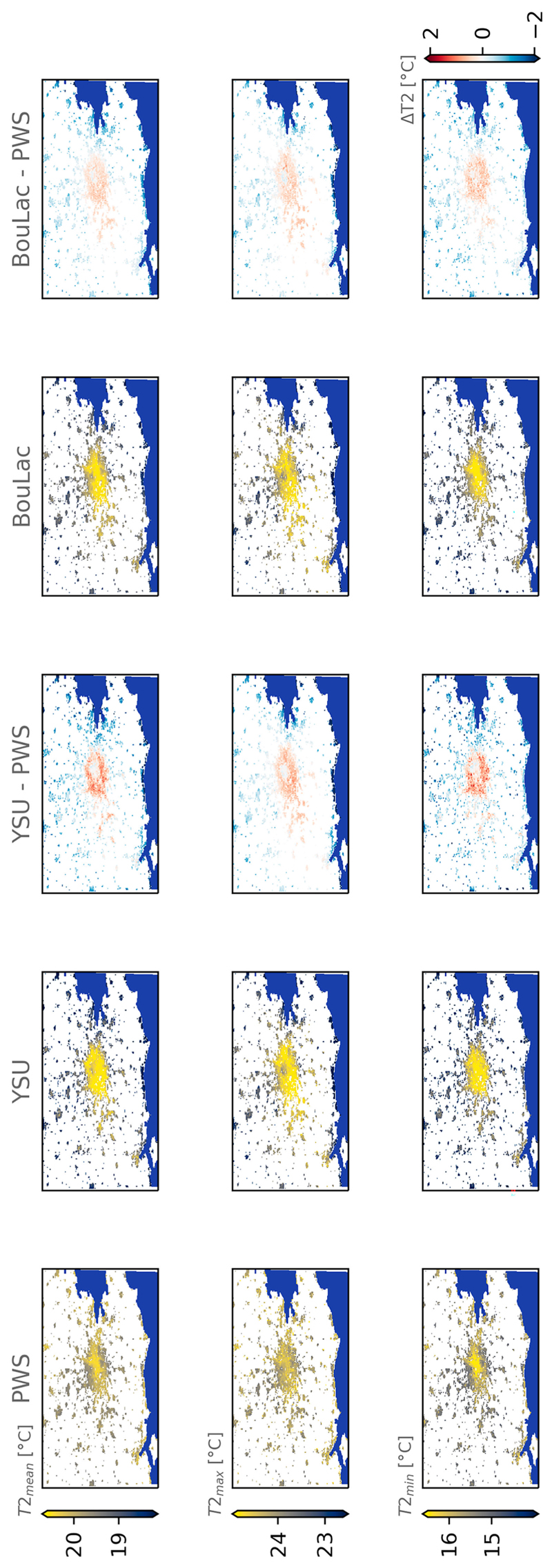
The random forest regressor leads to different bias corrections of the two WRF simulations parameterized with different turbulence schemes—YSU and BouLac—and with the BEP–BEM urban canopy model activated. This holds for average daily mean, minimum, and maximum temperatures (T2_mean_, T2_min_, and T2_max_) after the daily time-step bias correction. Compared to the predicted temperatures using the PWS data only (PWS), the bias-corrected products are hotter in the suburban areas of Greater London and cooler in the rural areas. The difference is more pronounced in YSU (see YSU 2 PWS). Grayed areas represent natural areas where the bias correction is not performed, and the sea is shown in dark blue. The same figures for the other regressors are given in Figs. C7–C11.

Finally, we find that the bias-corrected BouLac simulation corresponds spatially to predicted temperatures using PWSs more than YSU—something we find equally across all regressors (Figs. 6 and C7–C11). As an example, when comparing the average bias-corrected products using the time-step-trained random forest regressor, we can see that YSU’s urban heat is more homogeneously distributed than BouLac’s or the predicted temperatures from PWSs only. BouLac’s bias-corrected product shows stronger urban heat in central London compared to suburban areas, coherent with the predicted temperatures. Nonetheless, BouLac’s suburban areas are hotter by 0.5°–1.0°C than the predicted ones with PWSs only. This remains less pronounced than in YSU. Last, we can see that both bias-corrected products show similar trends when compared to the PWS-only predicted temperatures with hotter suburban areas and cooler secondary cities as well as coastlines. Again, this does not show which product between the PWS-only predicted temperatures and the bias-corrected products is better since we do not evaluate this here.

These results show that bias correction of modeled air temperatures changes their spatiotemporal distributions. When focusing on the potential impact bias correction may have in estimated urban heat impact on urban health, we find that using the random forest regression trained at each daily time step leads to an increased average population weighted temperature by 0.77°C in the YSU case and by 1.24°C in the BouLac case. Raw model outputs are thereby lowering the impact of heat on the urban population.

## 4 Discussion

In this study, we argue that the joint use of data from crowdsourced PWSs and UCMs can add value to urban climate research and in particular to urban climate impact research. This is supported by two major outcomes of our case study focused over London during the summer 2018. First, we showed that evaluation of urban climate simulations using PWSs enables the detection of spatially varying systematic biases in urban areas related to the UCMs’ parameterization, which are not detectable using only official weather stations. Second, we demonstrated that PWSs, combined with detailed morphological data derived from LCZ maps, can be used to derive a spatially varying bias correction via commonly used machine learning regressors. This latter point has major implications for urban climate impact research—and especially future urban climate impact studies—as we hereby propose the first bias-correction technique that considers the existence of a nonlinear spatially heterogeneous bias in modeled urban climates.

Of course, using PWSs for evaluating UCM simulations should always cautiously be considered because of the lower accuracy of PWSs and the potential uncertainties related to user-driven mistakes in the setup of their PWSs (e.g., indoor sensors instead of outdoor, poor shading conditions, height of the sensor). However, reliable tools have now been developed since the first use of PWSs for model evaluation by Hammerberg et al. (2018) to filter dubious measurements out [e.g., CrowdQC from Napoly et al. (2018) or CrowdQC+ by Fenner et al. (2021)], thus making PWS observations increasingly reliable. This does not resolve the question of the representativity of measurements, that is, “how is one PWS measurement representative of the simulated urban pixel?” Yet, the increasing density of PWSs in the urban environments begins to alleviate this uncertainty—despite a recognized unequal distribution of PWSs among a variety of environmental, socioeconomic, and demographic indicators (Brousse et al. 2023). For example, Venter et al. (2020) found that a density of one PWS per square kilometer is optimal for predicting seasonal air temperature in Oslo. Dense PWS networks hence permit the detection of systematic biases that would otherwise pass undetected. Therefore, to support the development of PWSs as a source of urban weather observations for model evaluation, urban climate scientists should identify an optimal density of PWSs for UCM evaluation, to define which cities need urban weather observations, and to start instigating common frameworks and standards.

We consider our study to be innovative and supportive of future advances in the field because it is the first bias-correction technique in urban environments that considers that the accuracy of the simulated UHI is spatially heterogeneous due to the complexity of the urban surfaces and the lack of a linear correlation between urban environmental archetypes and temperatures at local scales. Aided by the expanding fields of crowdsourcing weather observations through PWSs, machine learning, and potentially deep learning, we infer that our work should serve as the basis of future research that would try to improve the bias correction of urban climate models using PWSs. For instance, we did not find any machine learning regressor to be more efficient at predicting the model bias. This could be explained by the rather restricted set of covariates we used for training the regressors as well as the coarse horizontal resolution of 1 km at which the covariates were aggregated to be consistent with the model’s spatial resolution. Higher spatial resolutions and more specific satellite Earth observations could be used to improve regressors’ performance, following up on the work by Venter et al. (2021), for example. When modeling the near-surface UHI, their regressor achieved similar performances as ours, with an RMSE of 1.05°C and Pearson’s *r*^2^ of 0.23; it is important to note that these are the performance metrics for predictions of temperature rather than urban climate model bias. Although the common use of model’s input parameters and Earth observations as covariates could be beneficial, particular attention should be given to the choice of Earth observations since these should not be decorrelated to the model’s physics and dynamics as the purpose would remain the bias correction.

Independent of the set of covariates used in this study, we found that the regressors’ performances greatly improved when trained over a certain number of PWSs (more than ~90) before plateauing. Because of this, future research should try to investigate how machine learning regressors could benefit from unfiltered PWS data and other PWS data sources. Interestingly, we found that official sources of data like MIDAS were detrimental to the regressors, potentially because official weather stations tend to be placed in open fields or parks without surrounding built-up areas to increase measurement accuracies. This would explain why our regressors tended to further increase the systematic cool bias when using only MIDAS stations for training as parks are typically cooler at night and on average than more urbanized areas where PWSs are located. In addition, we found that training regressors at the daily time step did not outperform training with the summertime-mean average. Regressors could therefore gain in performance by adding a temporal component to the covariates. Following up on this idea, the recent work by Zumwald et al. (2021) tried predicting the near-surface air temperature in Zurich for 30 June 2019 out of ~650 Netatmo PWSs’ measurements during the preceding week. Their set of covariates consisted of spatial Earth observations as well as 35 meteorological predictors that were all derived from one of the official automatic weather stations. The latter predictors helped train the model to recognize how the temperature measured at each PWS location was related to the meteorological variables measured at the automatic weather stations. Their predictions at hourly time steps achieved reasonable performances with RMSEs around 1.70°C. Bias correction of UCM simulations could hence be improved by incorporating temporally explicit meteorological observations from official weather stations. Notwithstanding, this would require extensive investigation of the area down to which each official station is representative for training the regressors. More geographically oriented machine learning regressors, like the geographical random forests (Georganos et al. 2021), could also help integrate these spatial heterogeneities for an improved bias correction.

In general, we support the use of quality-checked PWS observations for bias correction of urban climate simulations. As shown in this case study, model outputs prior to any bias correction could lead to under- or overestimation of urban heat impact on public health. We indeed find that for the summer 2018 in London, average population weighted temperatures—which are directly correlated to heat-related mortality—were higher after bias correcting the model outputs. This suggests that there could be a higher urban heat-related mortality during this period that would not be captured without bias correction. This simple example shows that bias correction of urban climate simulations could have important implications for calculating the exposure of urban citizens to heat or estimating the urban heat-related mortality. Although preferring bias-corrected model outputs to predicted urban air temperatures from Earth observations for present-day urban heat impact studies is not covered in this study—and must be further explored—we still argue that bias correction should be done prior to any urban heat impact studies that imply using climate model outputs. This argument is especially valid for future climate projections at urban scale, and we encourage future research to investigate how to transfer present urban bias-correction coefficients to simulated future urban climates. Doing so, bias-corrected simulations could help targeting areas where heat mitigation or adaptation strategies could be more beneficial as their efficiency is dependent on their location and scales of implementation (Yang and Bou-Zeid 2019; Broadbent et al. 2022). We also suggest that our methods could be extended to other fields of urban climatology and urban air quality. Several devices already offer the possibility to obtain information on air quality, precipitation, or wind speed, to name a few (de Vos et al. 2020). Hence, bias correction of regional climate models’ outputs using crowd-sourced data should not be restricted only to air temperatures.

## 5 Conclusions

We demonstrate that the higher density of quality-controlled data from personal weather sensor measurements of temperatures in cities like London is beneficial for urban climate model evaluation. We then show that PWSs could be helpful for bias correcting modeled temperatures using a set of machine learning statistical regressors. We did not observe tangible differences in performance of the regressors to predict the bias at various locations. A minimum of ~24% of the total sample size of PWSs (96 stations of the 402 used in this study) was required to efficiently train our regressors; official weather sources like MIDAS were detrimental to the urban bias correction, probably because of site specificities. Our work has important implications for urban climate impact studies that would make use of urban climate model outputs.

## Supplementary Material

Appendix

## Figures and Tables

**Table 1:** Thermal and radiative parameters per LCZ based on Stewart et al. (2014). Road parameters are considering a mixture of asphalted and concrete road pavements and grass.

	Heat capacity (J m^−3^ K^−1^)				Thermal conductivity (J m^−1^ s^−1^ K^−1^)				Albedo				Emissivity		
	Roof	Wall	Road		Roof	Wall	Road		Roof	Wall	Road		Roof	Wall	Road
LCZ 1	1.80 × 10^6^	1.80 × 10^6^	1.75 × 10^6^		1.25	1.09	0.77		0.13	0.25	0.15		0.91	0.90	0.95
LCZ 2	1.80 × 10^6^	2.67 × 10^6^	1.65 × 10^6^		1.25	1.50	0.73		0.18	0.20	0.16		0.91	0.90	0.95
LCZ 3	1.44 × 10^6^	2.05 × 10^6^	1.63 × 10^6^		1.00	1.25	0.69		0.15	0.20	0.18		0.91	0.90	0.95
LCZ 4	1.80 × 10^6^	2.00 × 10^6^	1.54 × 10^6^		1.25	1.45	0.60		0.13	0.20	0.20		0.91	0.90	0.95
LCZ 5	1.80 × 10^6^	2.00 × 10^6^	1.50 × 10^6^		1.25	1.45	0.62		0.13	0.25	0.20		0.91	0.90	0.95
LCZ 6	1.44 × 10^6^	2.05 × 10^6^	1.47 × 10^6^		1.00	1.25	0.60		0.13	0.25	0.21		0.91	0.90	0.95
LCZ 7	2.00 × 10^6^	7.20 × 10^5^	1.38 × 10^6^		2.00	0.50	0.51		0.15	0.20	0.24		0.28	0.90	0.92
LCZ 8	1.80 × 10^6^	1.80 × 10^6^	1.80 × 10^6^		1.25	1.25	0.80		0.18	0.25	0.17		0.91	0.90	0.95
LCZ 9	1.44 × 10^6^	2.56 × 10^6^	1.37 × 10^6^		1.00	1.00	0.55		0.13	0.25	0.23		0.91	0.90	0.95
LCZ 10	2.00 × 10^6^	1.69 × 10^6^	1.49 × 10^6^		2.00	1.33	0.61		0.10	0.20	0.21		0.91	0.90	0.95

**Table 2:** Hyperparameter tuning used by each regressor.

Model	Parameters dictionary
Linear	‘normalize’: False
Ridge	‘alpha’: 1, ‘normalize’: True, ‘random_state’: 42, ‘solver’: ‘lsqr’, ‘tol’: 0.01
Lasso	‘alpha’: 1, ‘normalize’: False, ‘random_state’: 42, ‘selection’: ‘random’, ‘tol’: 1 × 10 ^−10^
Random forest	‘max_features’: ‘sqrt’, ‘min_samples_leaf’: 11, ‘min_samples_split’: 2, ‘n_estimators’: 400, ‘random_state’: 42
Gradient boosting	‘learning_rate’: 0.2, ‘max_depth’: 3, ‘max_features’: ‘sqrt’, ‘min_samples_leaf’: 10, ‘min_samples_split’: 22, ‘n_estimators’: 200, ‘random_state’: 42, ‘subsample’: 0.2

**Table 3:** Average of all performance metrics calculated at each MIDAS official weather stations for hourly air temperature at 2 m for the summer period (1 Jun 2018–31 Aug 2018). Urban stations are stations located in a pixel classified as an urban LCZ in WRF and rural stations are located in other natural land use/land cover.

	BouLac						YSU				
	RMSE	MAE	MB	*r* ^2^	*r*		RMSE	MAE	MB	*r* ^2^	*r*
All	2.33	1.82	−0.56	0.77	0.86		2.31	1.83	−0.57	0.79	0.88
Urban	2.42	1.88	−0.73	0.76	0.86		2.42	1.92	−0.93	0.77	0.87
Rural	2.32	1.81	−0.53	0.78	0.86		2.28	1.81	−0.50	0.80	0.88

## Data Availability

The simulations done in this research were performed using the WRF Model v4.3 (https://github.com/wrf-model/WRF.git). The scripts and WRF name-lists used in this study are accessible at https://github.com/oscarbrousse/JAMC_BiasCorrection_PWS/. The related outputs presented in this research are available upon reasonable request addressed to the corresponding author.

## Significance Statement

Urban climate simulations are subject to spatially heterogeneous biases in urban air temperatures. Common validation methods using official weather stations do not suffice for detecting these biases. Using a dense set of personal weather sensors in London, we detect these biases before proposing an innovative way to correct them with machine learning techniques. We argue that any urban climate impact study should use such a technique if possible and that urban climate scientists should continue investigating paths to improve our methods.
