# Supplementary material for: Spatially Explicit Correction of Simulated Urban Air Temperatures Using Crowdsourced Data
Source: J Appl Meteorol Climatol. Author manuscript; Available in PMC 2024 Jun 13. (PMC7616100; doi:10.1175/JAMC-D-22-0142.1)
Supplement: Appendix [file EMS196712-supplement-Appendix.pdf]

APPENDIX A

Model Sensitivity Testing over the Two Hottest Days of Summer 2018

Prior to running the 3-month simulation, we tested the model's sensitivity to a set of parameterizations to assess which model is the best performing model for the 3-month simulation. We perform the sensitivity in a progressive way; parameters are kept if beneficial and removed if detrimental. We chose to run the simulations over the two hottest days of the summer 2018 with one additional day as spinup time—from 25 to 27 July 2018—to see how the model is capable of accurately representing an extreme condition in terms of air temperature at 2 m—tested against official MIDAS automatic weather stations and personal Netatmo PWSs. The model was also tested for relative humidity and wind speed at 10 m at MIDAS locations where records were available. All wind speed measurements are converted from knots to meters per second.

We start from Heaviside et al. (2015) model's parameterization, who simulated the impact of urbanization on the local climate in the West Midlands in England but supplement the Coordination of Information on the Environment (CORINE) land-use/land-cover by the local climate zones classification instead since Brousse et al. (2016) compared both products and proved the added value of LCZ over Madrid. We chose the work by Heaviside et al. (2015) as a starting point since it also uses the BEP urban climate model, coupled to the WRF Model and is one of the only WRF simulations done over England.

From there, our simulations tested (i) the use of YSU, recently coupled to the BEP–BEM (Hendricks et al. 2020), instead of Bougeault–Lacarrère; (ii) the use of the more complex land surface scheme Noah-MP in its default parameterization instead of the default Noah land surface model; (iii) the forcing by ERA5 reanalysis data at 25-km horizontal resolution instead of ERA-Interim; and (iv) the reduction of soil moisture by 50% and its increase by 200%, following suggestions provided by Martilli et al. (2021). We chose not to test the impact of urban canopy parameters in this case to keep our simulations standardized and universally coherent through the LCZ scheme. Their simulation used the same micro, clouds, convection, and radiation physics as ours.

We found that all steps taken from the original parameterization by Heaviside et al. (2015) were beneficial to the model's performance. Through an intermediate simulation where we tested again the BouLac turbulence scheme after step iii, we found that YSU was still performing better.

APPENDIX B

Sensitivity of Machine Learning Regressors to Data Quality and Quantity

Before running our bias correction and our bootstrapping, we needed to evaluate the degradation in performance of all the regressors in relation to the quantity of data available for training. In this way, we could ascertain that the chosen amount of 80% for running the bootstrapping procedure was not detrimental to the regressors' performances. Additionally, despite the fact that official weather data coming from MIDAS are usually coming from open fields like airports or parks, we still chose to test how our model performs if only these data were available for bias correction, thereby ensuring that the use of the dense network of PWSs is useful for bias correction. To test this, we trained all the regressors over both WRF boundary layer conditions to bias correct the summertime average daily mean, minimum, and maximum temperatures. This means that we are testing the ability of the regressors to predict the bias at certain PWS locations to correct the modeled temperature. In this case, we evaluate the bias-corrected temperatures against the observed temperatures. We chose not to run over daily time steps as this would be too computationally expensive.

We followed a bootstrapping procedure, where 20% of the PWS temperature data were randomly selected and kept for testing the regressors' performance. Random samples with increasing ratios of the remaining 80% of PWS temperature data and covariates were used to train the regressors 25 times. We ensured that the randomly sampled 20% and ratios are kept constant between regressors. We first started with 1% of the remaining 80% and increased the ratio by steps of 1% to 10% of the remaining 80%. Steps of 10% were then used until reaching 90% of the remaining 80%. We chose to use these steps as we expect our regressors' performance to rapidly increase with a low amount of data before plateauing with a greater amount of data. Then, to test the added value of urban PWS density and data, we trained the same regressors over the modeled bias at the 10 urban MIDAS station locations and evaluated the bias correction against the 20% of the PWS data kept for evaluation at each bootstrapping step. As a comparison, we also evaluated the WRF output prior to bias correction against the same 20% of PWS temperature data at each

Unauthenticated | Downloaded 06/05/24 10:06 AM UTC

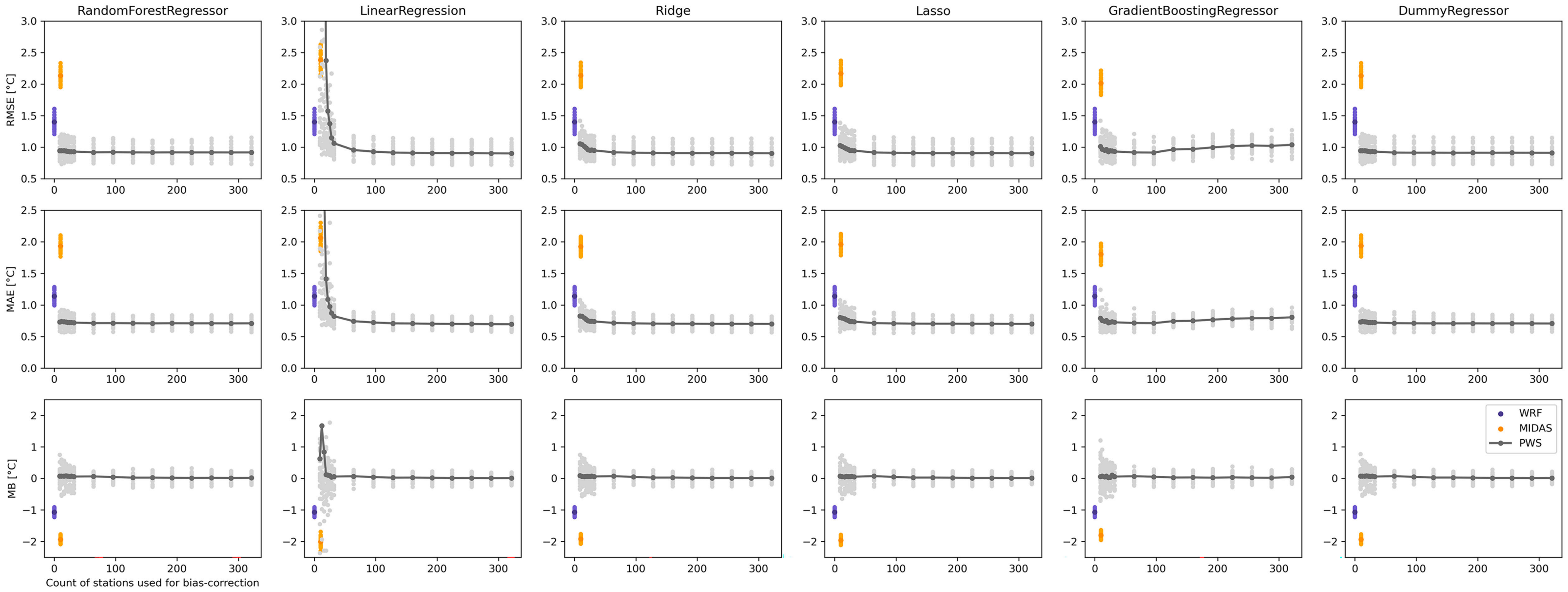

FIG. B1. Regressors' performance for bias correction of the summer average daily minimum air temperature depending on the amount of weather stations used for training. The performance is evaluated with the MAE (°C), RMSE (°C), and mean bias (MB; °C). Blue dots represent the WRF Model's performance prior to bias correction, orange dots are the performance of the WRF Model after bias correction using MIDAS official weather stations, and gray dots are the performance of the WRF Model after bias correction using subsets of the available Netatmo personal weather sensors. Small lighter dots are representative of performances measured at each bootstrapping step (n = 25) and large darker dots are the average of all bootstraps. Here, the WRF Model was run with BouLac.

Unauthenticated | Downloaded 06/05/24 10:06 AM UTC

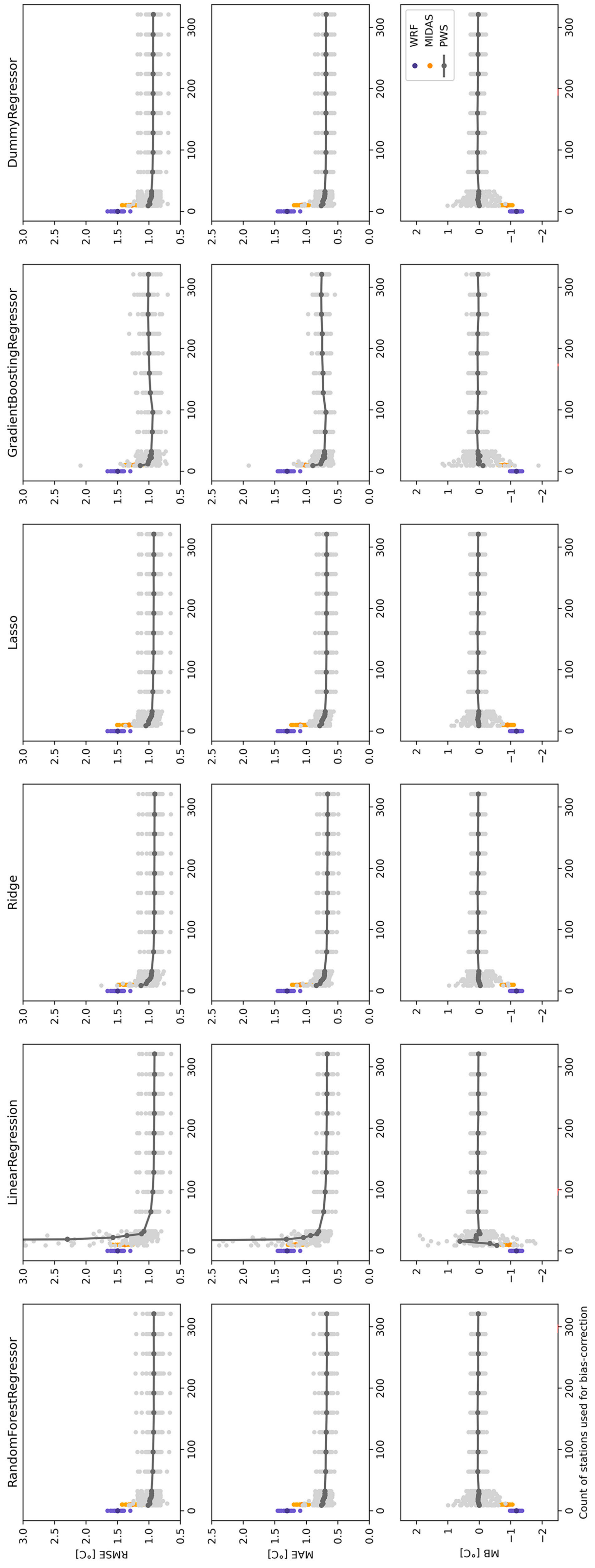

FIG. B2. As in Fig. B1, but for summer average daily mean temperatures.

Unauthenticated | Downloaded 06/05/24 10:06 AM UTC

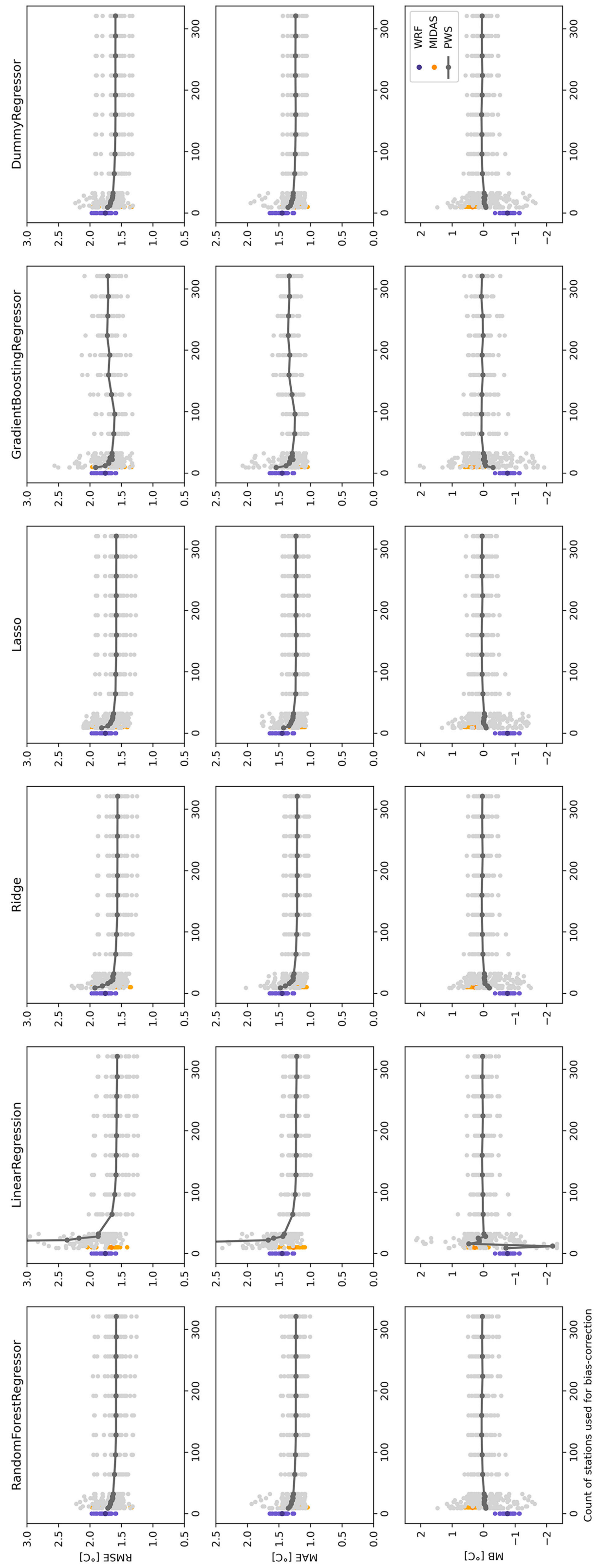

FIG. B3. As in Fig. B1, but for summer average daily maximum temperatures.

Unauthenticated | Downloaded 06/05/24 10:06 AM UTC

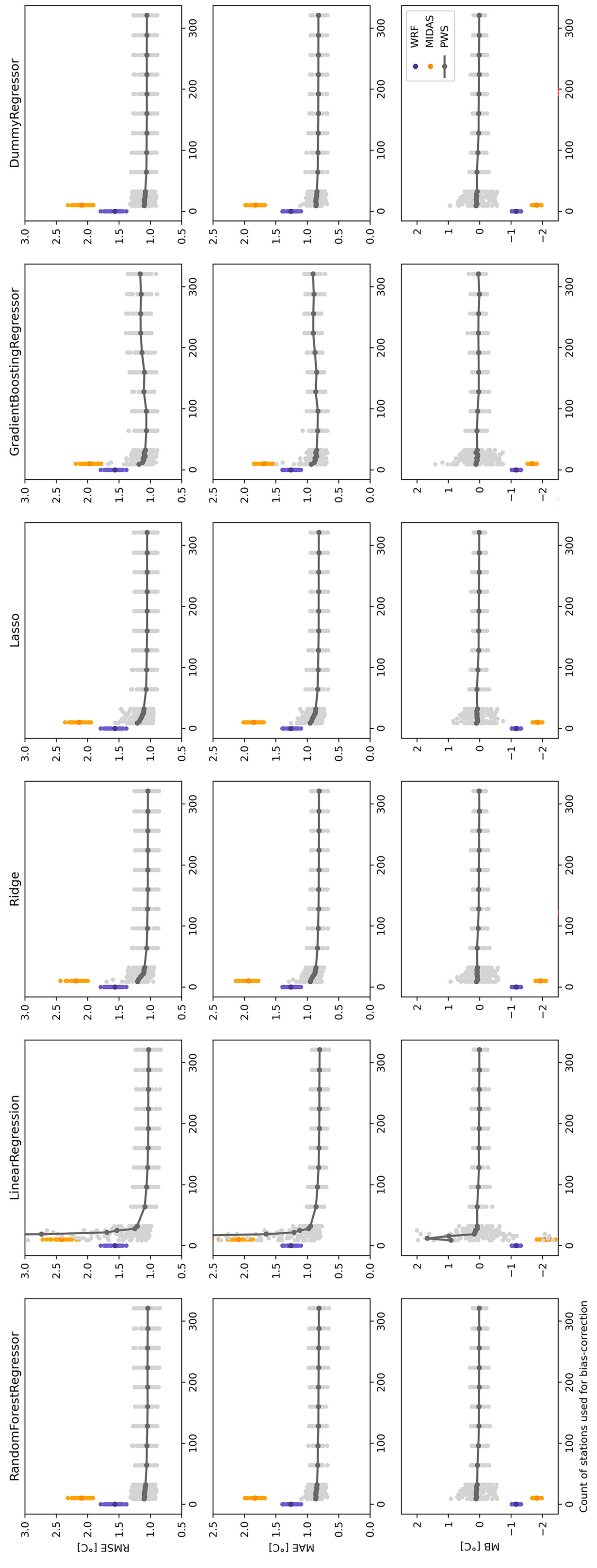

FIG. B4. As in Fig. B1, but for the WRF Model using the YSU PBL scheme.

Unauthenticated | Downloaded 06/05/24 10:06 AM UTC

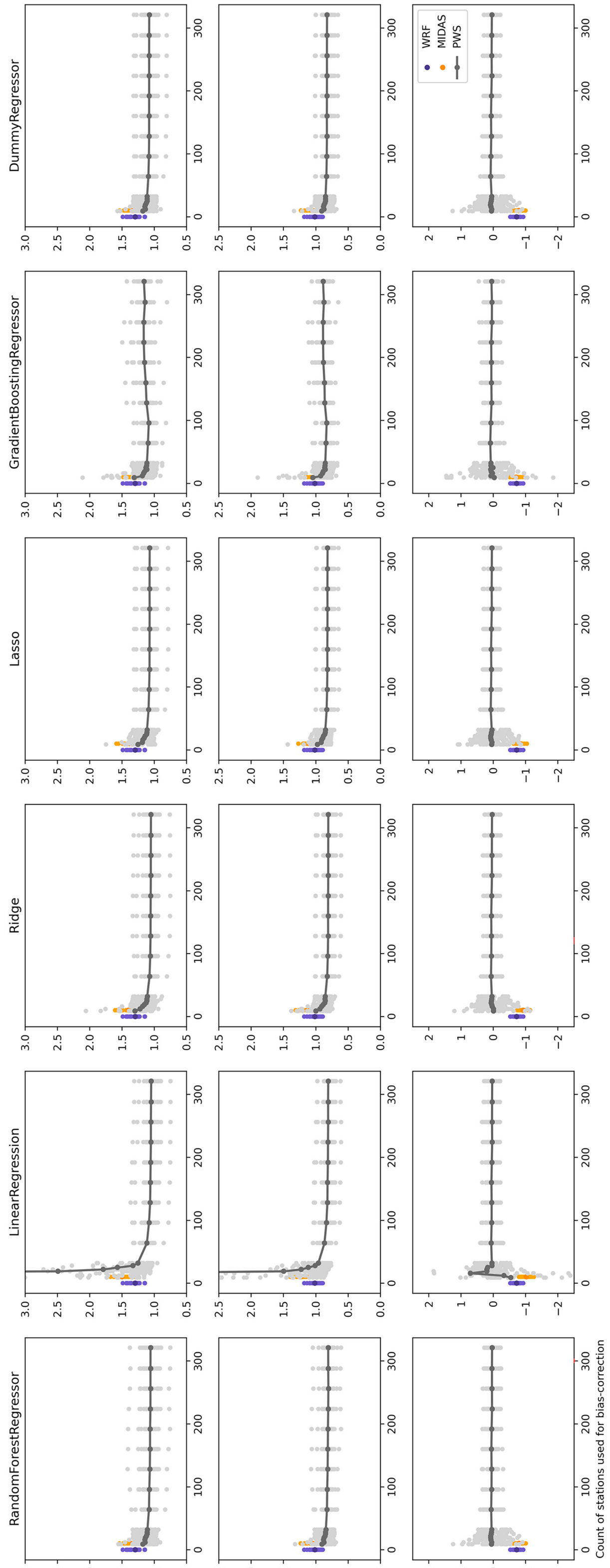

FIG. B5. As in Fig. B4, but for summer average daily mean temperatures.

Unauthenticated | Downloaded 06/05/24 10:06 AM UTC

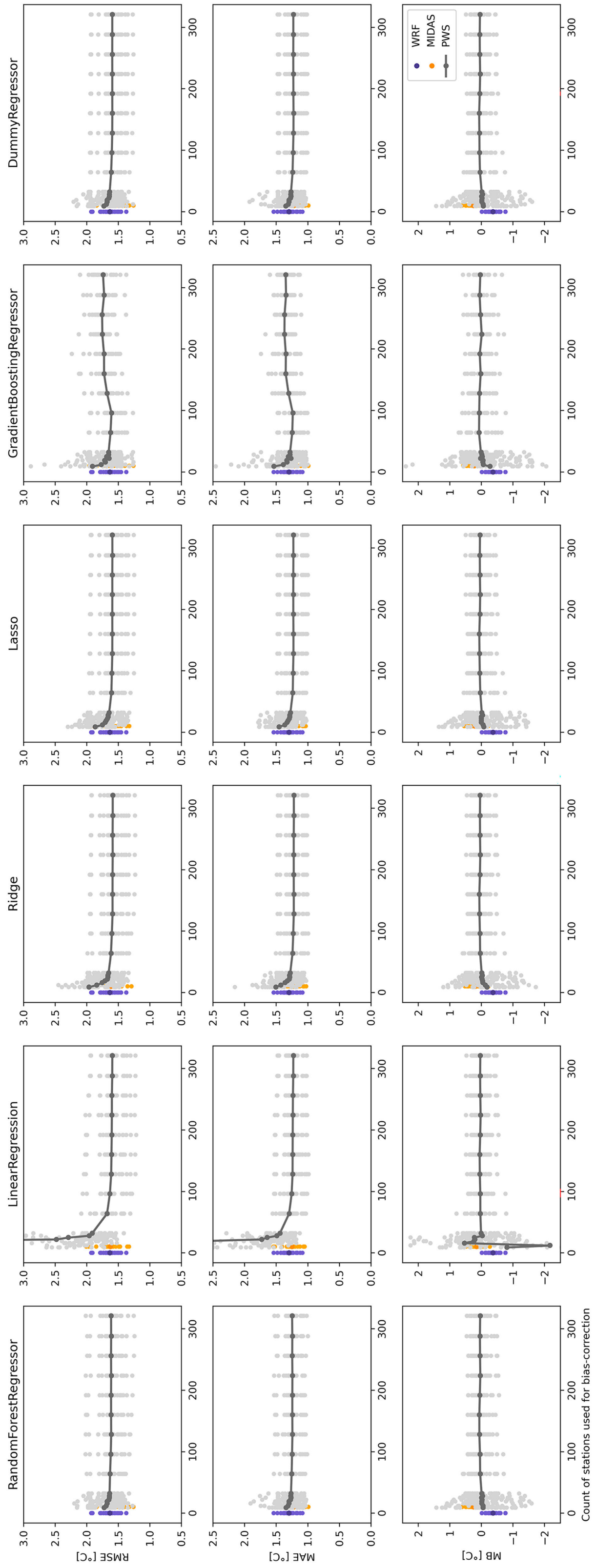

FIG. B6. As in Fig. B4, but for summer average daily mean temperatures.

Unauthenticated | Downloaded 06/05/24 10:06 AM UTC

bootstrapping step to demonstrate the added value of bias correction using a certain number of PWSs.

We found that all regressors benefited from a greater amount of PWS data, which reduced the RMSE, the MAE, and the MB on average and also reduced the variability of performances between each bootstrap sample (Figs. B1–B6). Only gradient boosting showed a slightly deteriorated performance by having more than 30% of the 80% PWS data used for training (96 PWSs)—probably due to overfitting. Below a number of 40 PWSs, all models performed poorly. We also showed that training the regressors over official MIDAS data only led to a poor bias correction for both summertime average daily minimum and mean temperatures. For the maximum, no clear benefit was demonstrable, which was also the case with PWSs and could be explained by the lower UHII during hot hours of the day, as discussed in the manuscript. We argue that this general outcome is explicable by the standard location of MIDAS weather stations—typically located in open parks or fields—which would explain why the bias correction for minimum temperatures further increases the cool bias already existing in WRF. This supports the use of PWSs for bias correction of urban temperatures for two reasons: first, the need for a sufficiently dense network of weather stations in urban environments; second, the necessity of weather stations located in typical built-up environments to accurately represent the effect of built-up surfaces on the local climate.

APPENDIX C

Additional Figures and Tables

This section presents all figures that are not given in the main text (Figs. C1–C11; Tables C1 and C2).

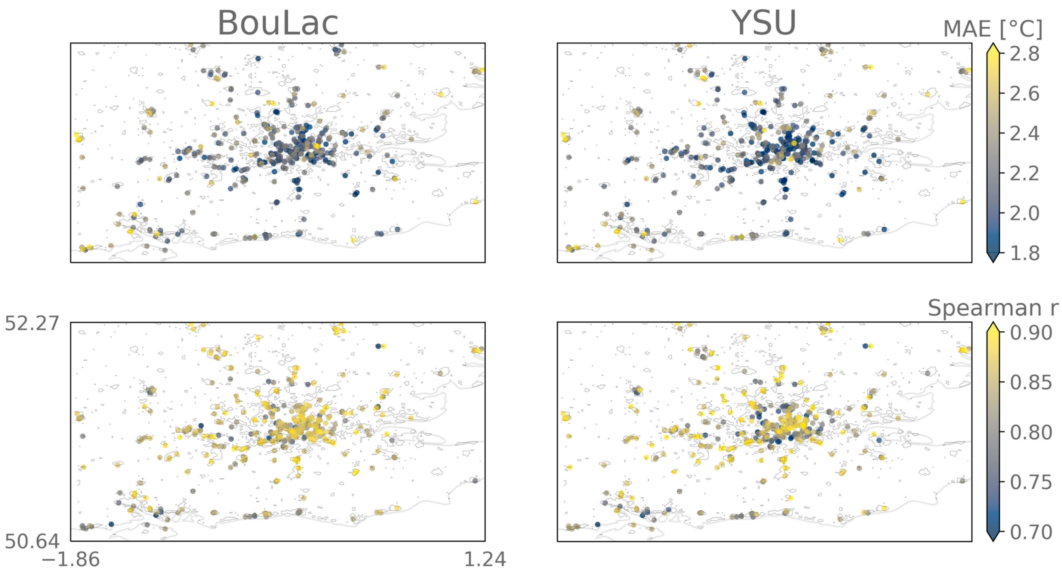

FIG. C1. As in Fig. 3, but for MAE and Spearman's r.

Unauthenticated | Downloaded 06/05/24 10:06 AM UTC

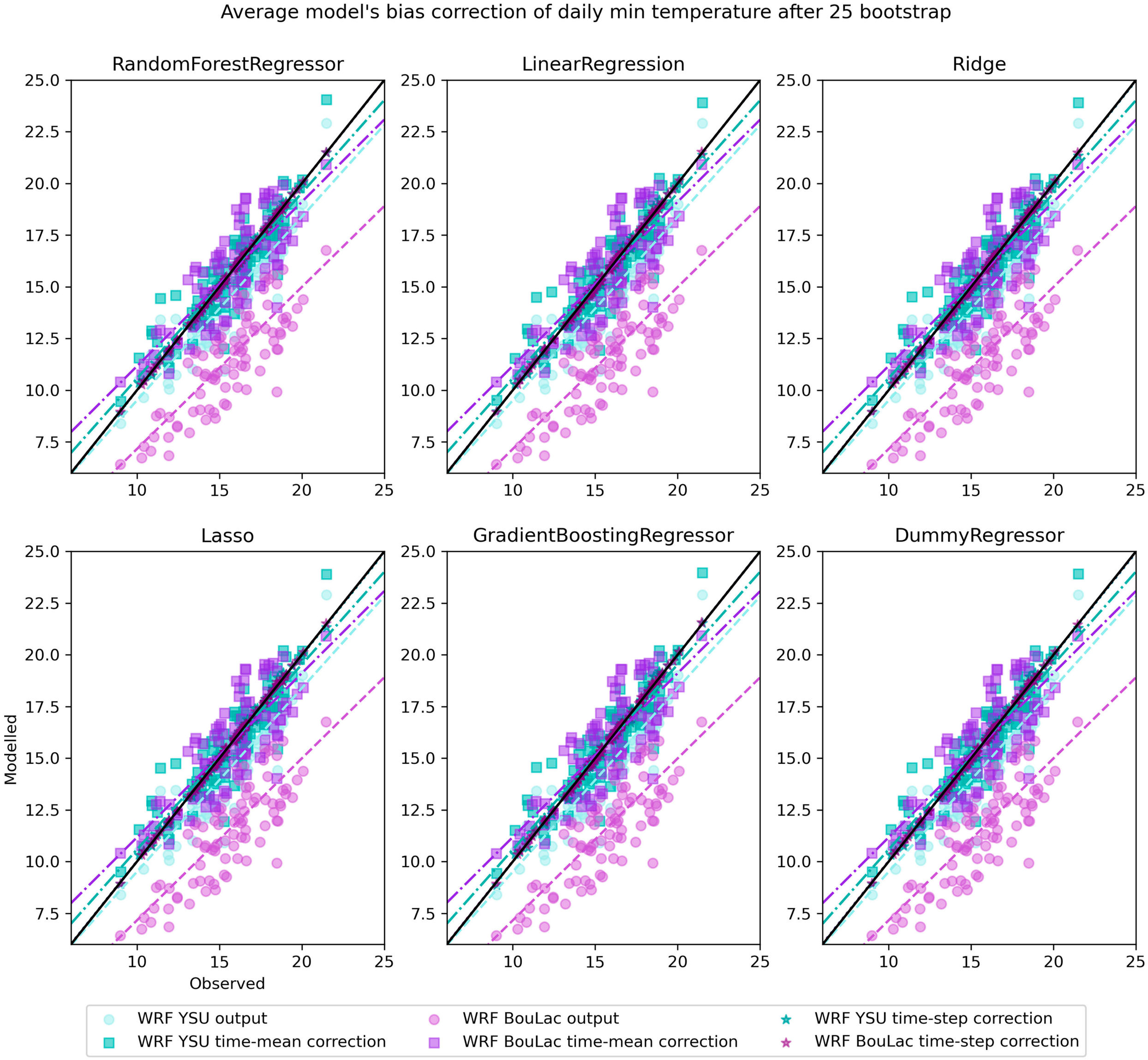

FIG. C2. Average modeled daily minimum air temperature at 2 m against observed at citizens' personal weather sensor locations show that all machine learning regressors perform a similar bias correction on average. In blue, modeled temperatures at 2 m are from the model simulation that used the YSU planetary boundary layer scheme before the bias correction (circles), after the summertime-mean bias correction (squares), and after the daily time-step bias correction (stars). In purple, the same values are given for the simulation which used the BouLac scheme. Dashed lines represent the least squares polynomial fitted lines and the black full line represents the identity line.

Unauthenticated | Downloaded 06/05/24 10:06 AM UTC

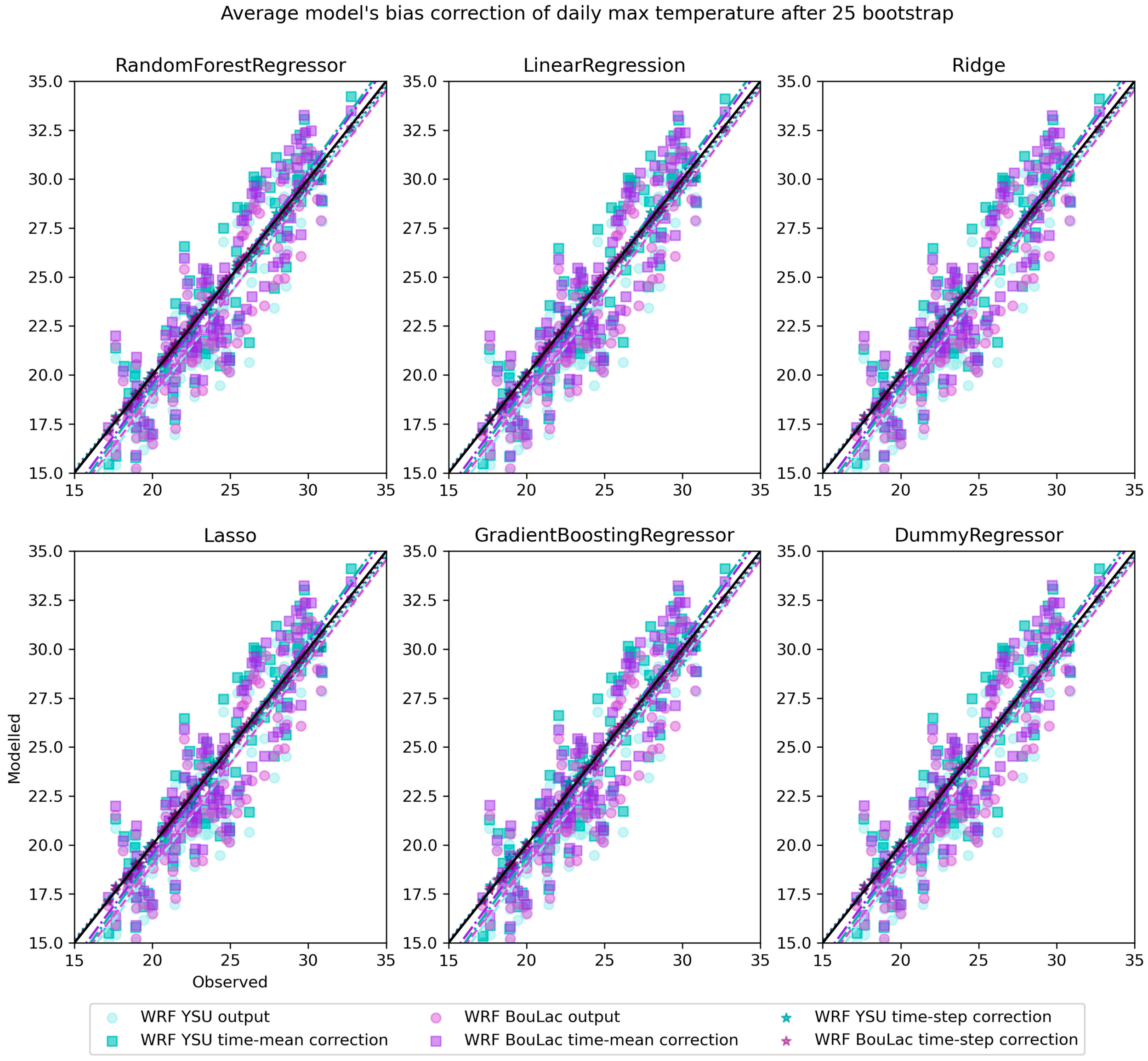

FIG. C3. As in Fig. C2, but for daily maximum temperatures.

Unauthenticated | Downloaded 06/05/24 10:06 AM UTC

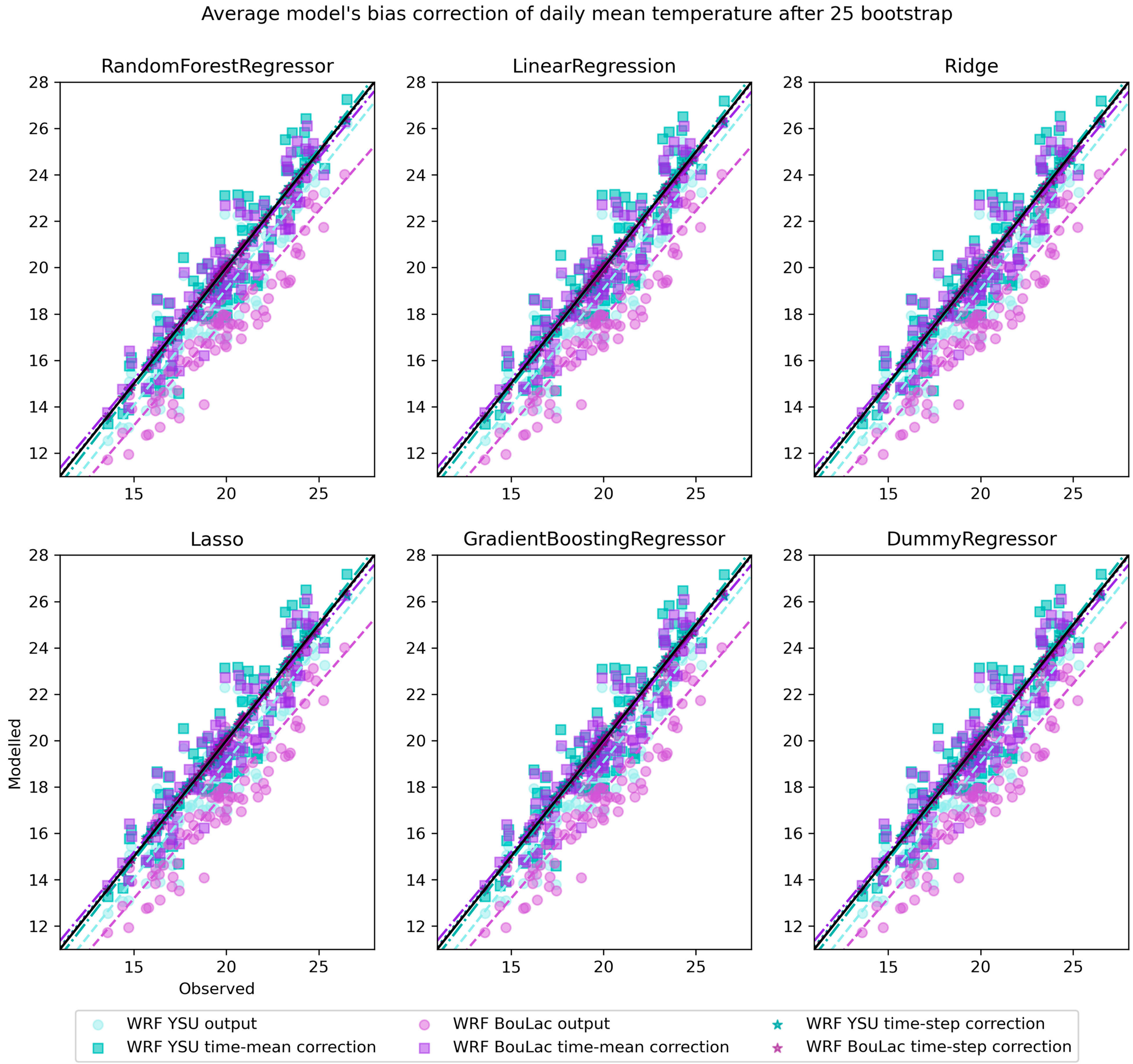

FIG. C4. As in Fig. C2, but for daily mean temperatures.

Unauthenticated | Downloaded 06/05/24 10:06 AM UTC

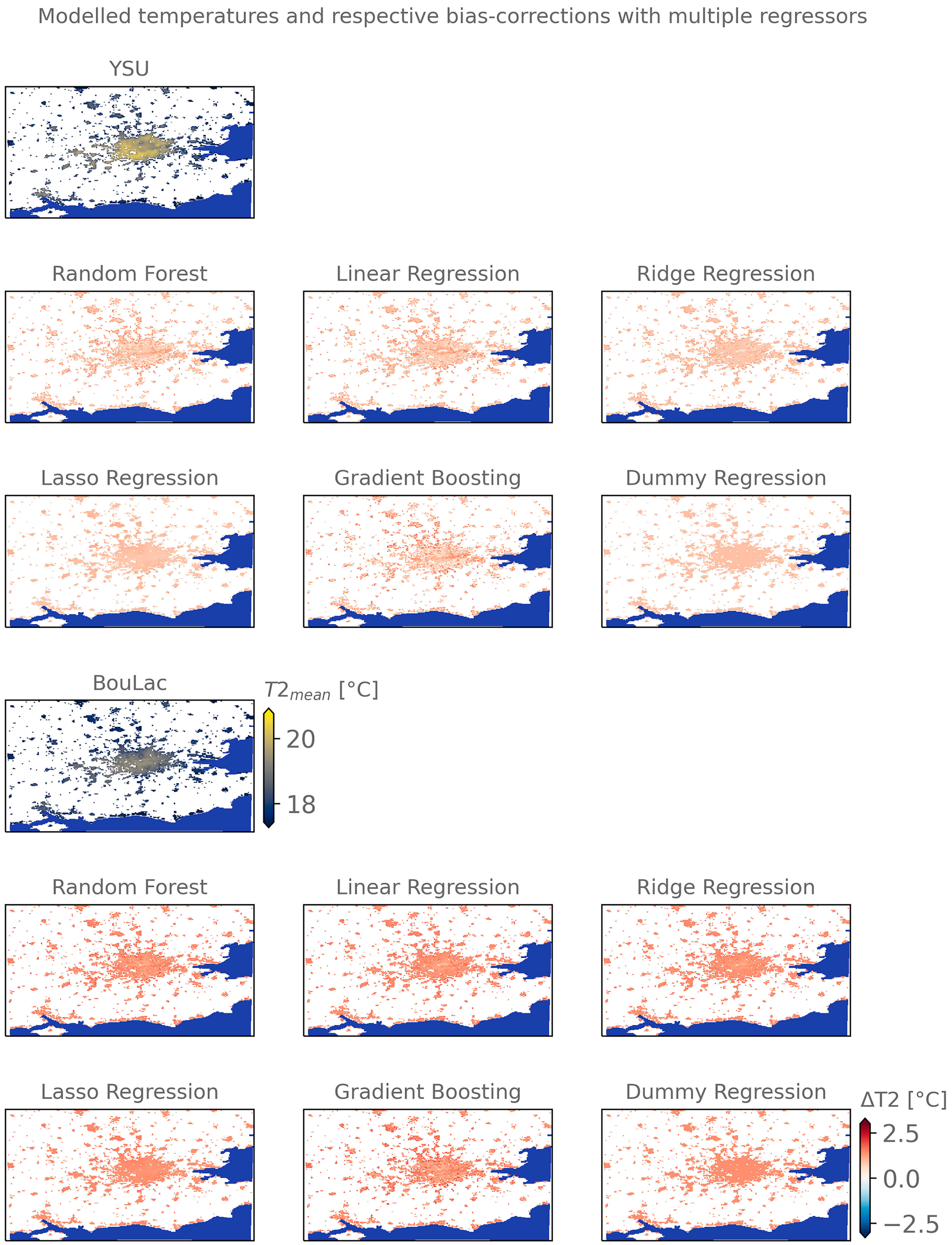

FIG. C5. As in Fig. 5, but for daily mean temperatures.

Unauthenticated | Downloaded 06/05/24 10:06 AM UTC

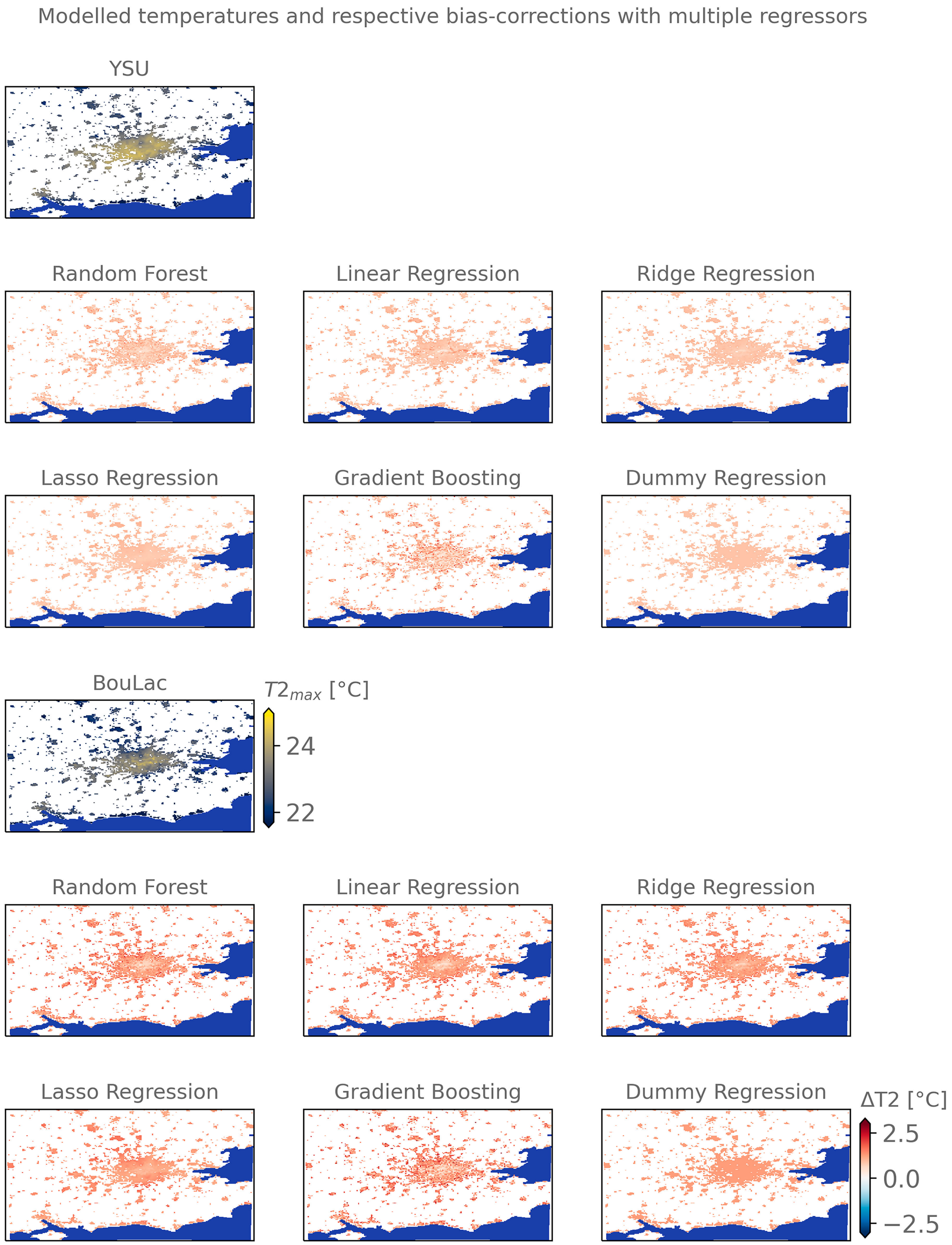

FIG. C6. As in Fig. 5, but for daily maximum temperatures.

Unauthenticated | Downloaded 06/05/24 10:06 AM UTC

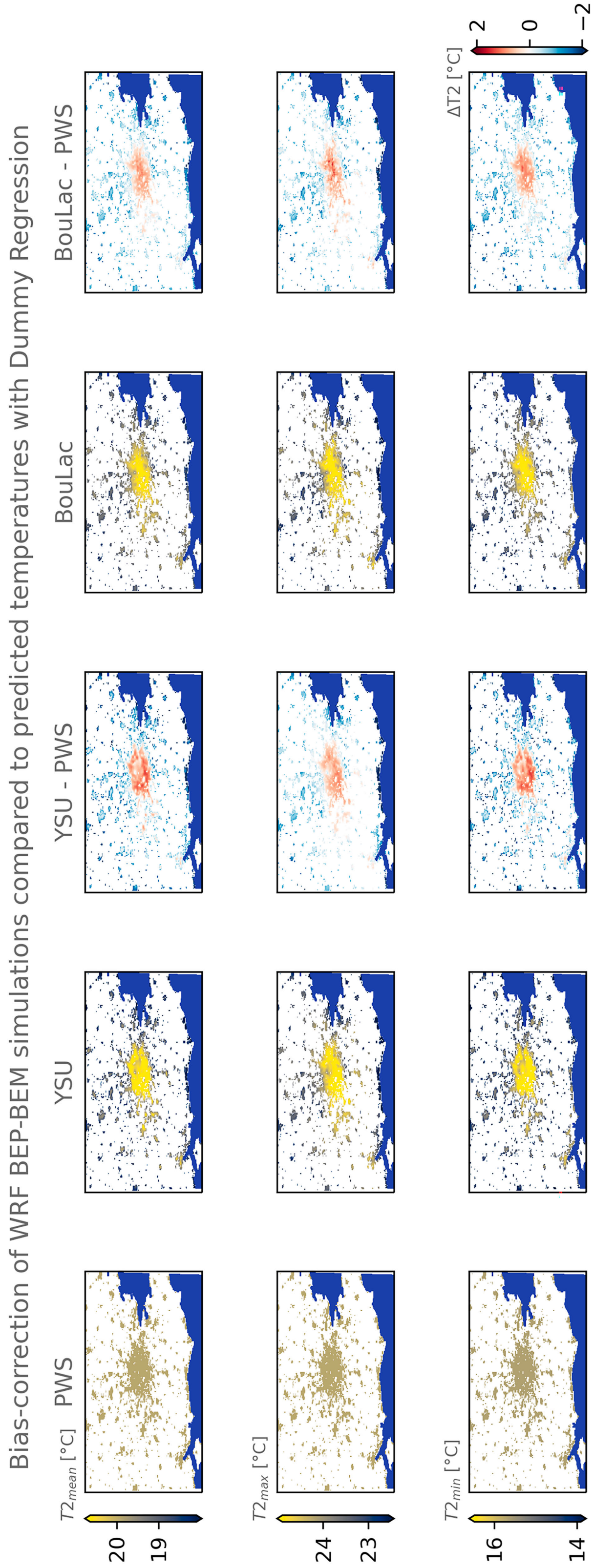

FIG. C7. As in Fig. 6, but for the dummy regression.

Unauthenticated | Downloaded 06/05/24 10:06 AM UTC

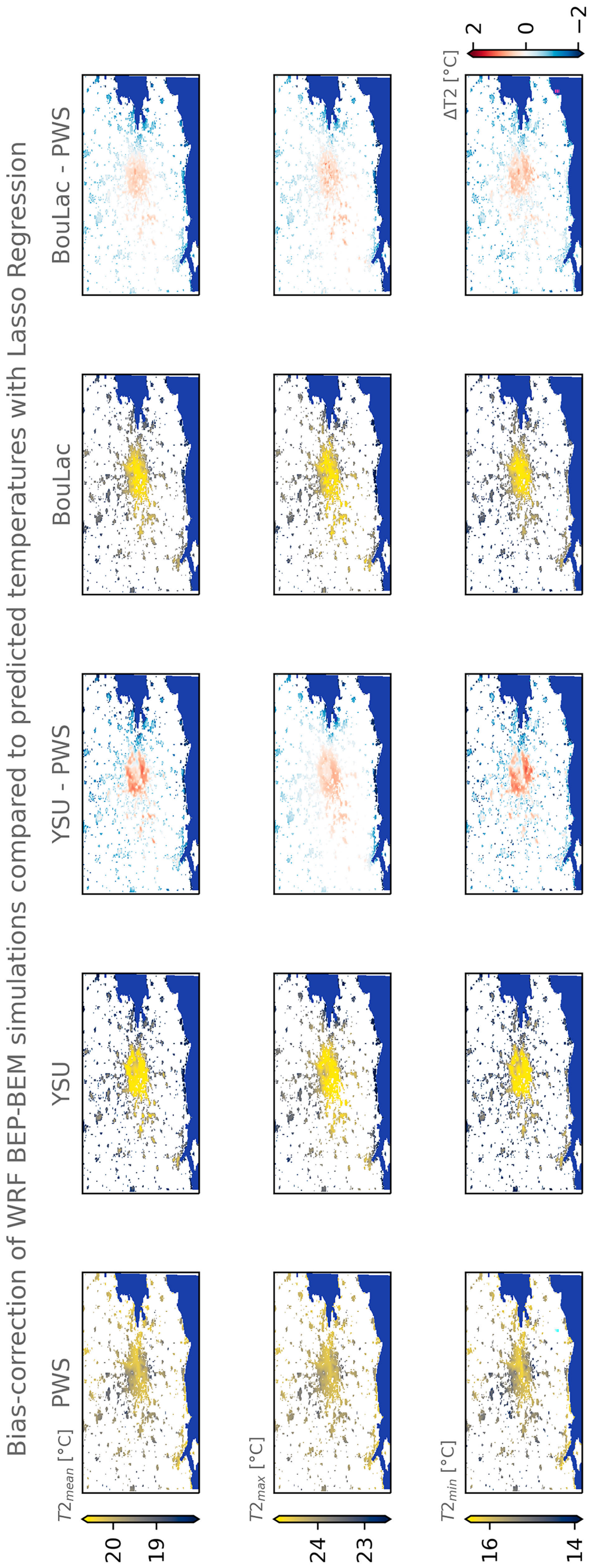

FIG. C8. As in Fig. 6, but for the Lasso regression.

Unauthenticated | Downloaded 06/05/24 10:06 AM UTC

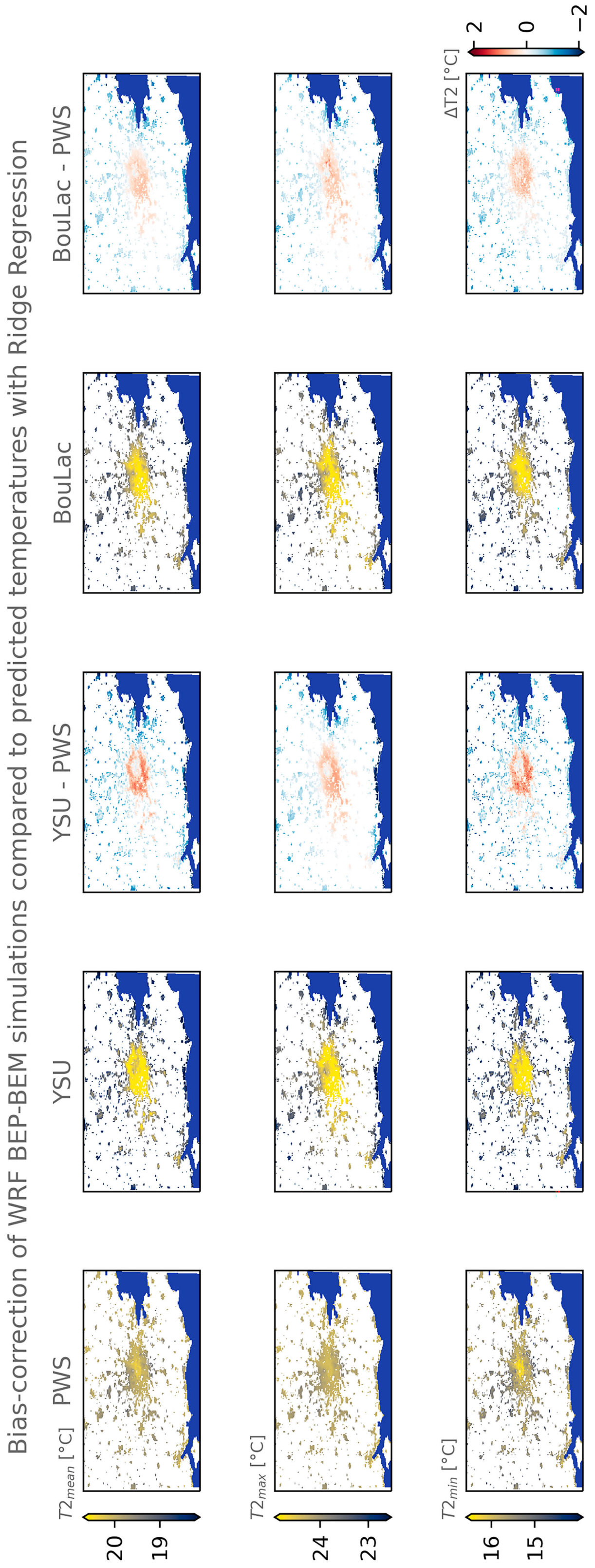

FIG. C9. As in Fig. 6, but for the Ridge regression.

Unauthenticated | Downloaded 06/05/24 10:06 AM UTC

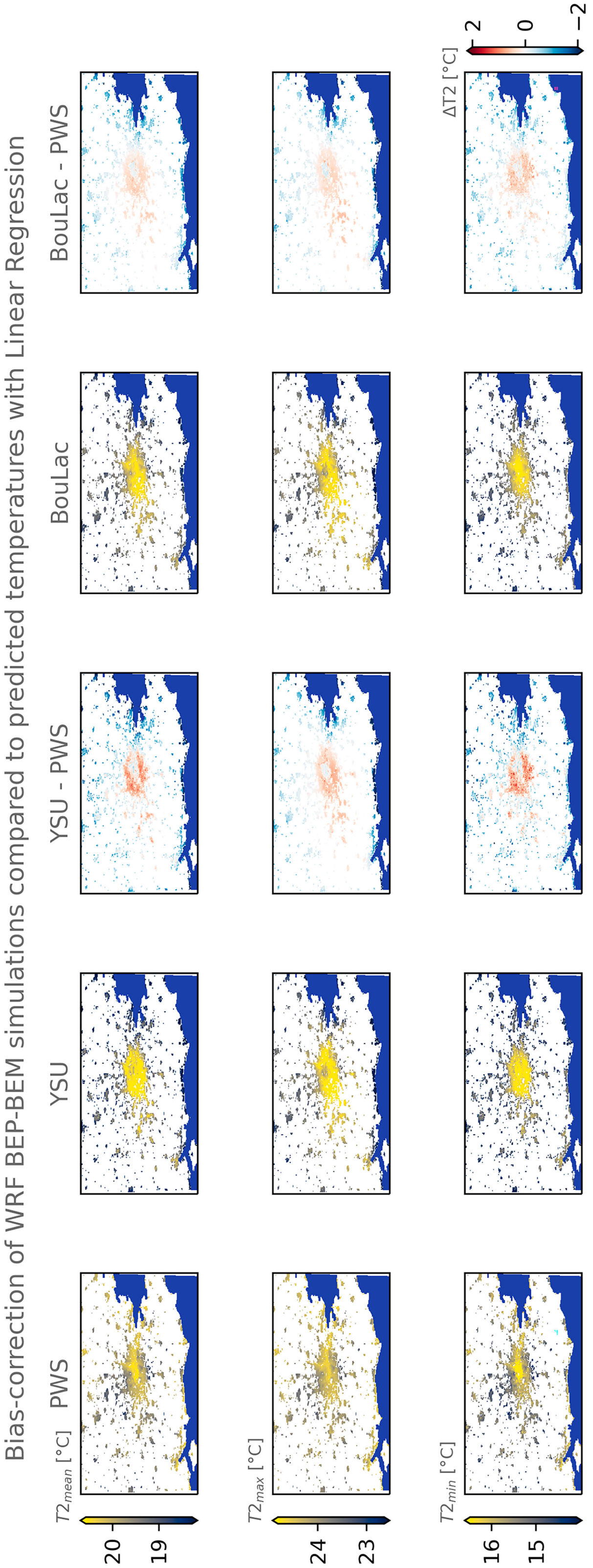

FIG. C10. As in Fig. 6, but for the linear regression.

Unauthenticated | Downloaded 06/05/24 10:06 AM UTC

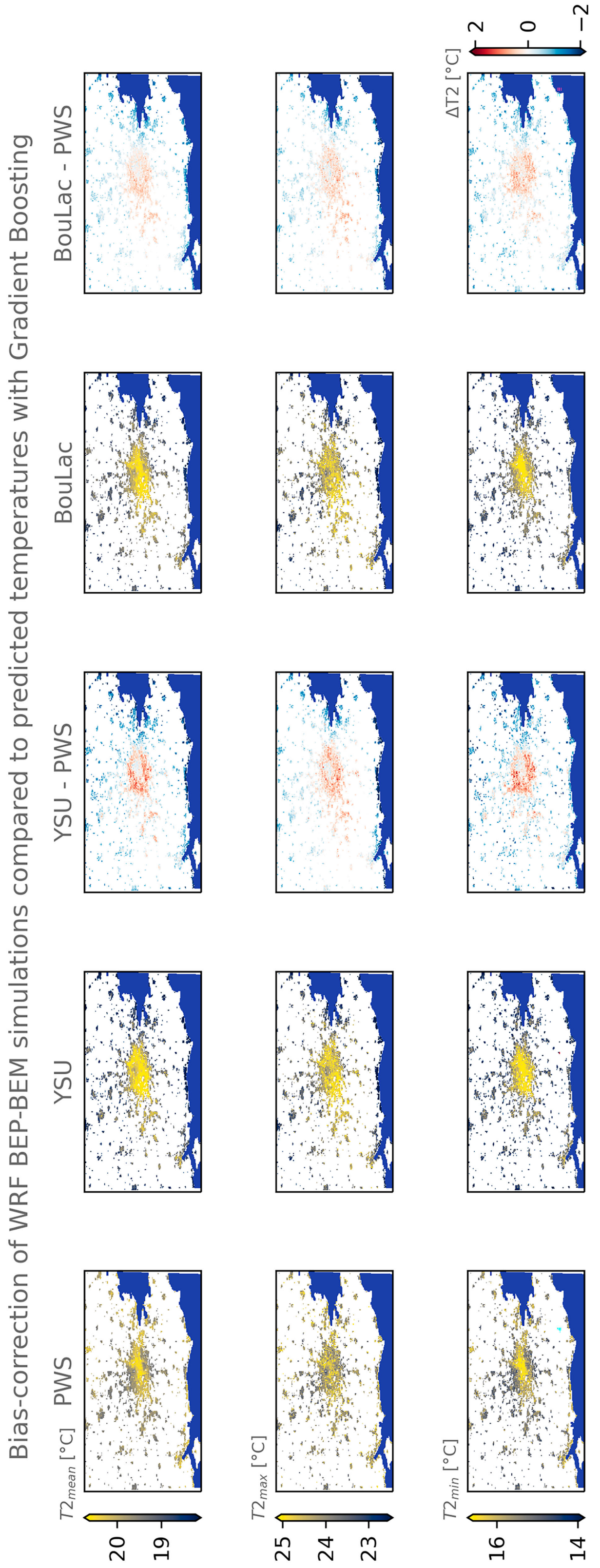

FIG. C11. As in Fig. 6, but for the gradient boosting regression.

Unauthenticated | Downloaded 06/05/24 10:06 AM UTC

TABLE C1. Performance metrics used in Fig. 4 for the model using BouLac prior to bias correction (WRF) and all the different regressors (random forest: RF; linear regression: LR; Ridge regression: RD; Lasso regression: LA; gradient boosting: GB; and dummy regression: DU). The different regressions are assigned a suffix: "avg" for regressions that were trained on the summertime-mean average of daily minimum, daily mean, or daily maximum temperatures and "tstep" for those that were trained with the temperatures at each daily time step.

	WRF	RF_{avg}	RF_{tstep}	LR_{avg}	LR_{tstep}	RD_{avg}	RD_{tstep}	LA_{avg}	LA_{tstep}	GB_{avg}	GB_{tstep}	DU_{avg}	DU_{tstep}
Mean													
RMSE	1.54	0.95	1.04	0.94	1.03	0.94	1.03	0.95	1.04	1.01	1.04	0.96	1.04
MAE	1.34	0.69	0.75	0.69	0.75	0.68	0.75	0.69	0.75	0.74	0.75	0.7	0.76
MB	−1.2	0.01	0.23	0	0.23	0	0.23	0	0.23	0	0.23	0.01	0.23
Pearson's r^2	0.11	0.09	0.07	0.09	0.07	0.1	0.07	0.1	0.07	0.06	0.06	0.11	0.08
Spearman's r	0.37	0.33	0.32	0.33	0.31	0.36	0.32	0.36	0.32	0.29	0.32	0.37	0.33
Min													
RMSE	1.42	0.93	0.94	0.92	0.93	0.92	0.93	0.92	0.93	1.01	0.96	0.92	0.94
MAE	1.15	0.72	0.73	0.71	0.72	0.71	0.72	0.71	0.73	0.79	0.74	0.71	0.73
MB	−1.08	0.01	0.02	0	0.02	0	0.02	0	0.02	0.04	0.02	0	0.02
Pearson's r^2	0.18	0.15	0.16	0.15	0.16	0.16	0.16	0.16	0.16	0.1	0.15	0.17	0.17
Spearman's r	0.46	0.42	0.43	0.43	0.42	0.44	0.43	0.44	0.43	0.34	0.41	0.46	0.44
Max													
RMSE	1.78	1.6	1.81	1.58	1.8	1.57	1.8	1.59	1.8	1.65	1.82	1.6	1.82
MAE	1.48	1.24	1.33	1.22	1.32	1.22	1.31	1.23	1.32	1.28	1.35	1.24	1.33
MB	−0.79	0	0.52	0	0.52	0	0.53	0.01	0.52	0	0.51	0.01	0.53
Spearman's r	0.08	0.07	0.02	0.08	0.02	0.09	0.02	0.08	0.02	0.05	0.01	0.08	0.03
Spearman's r	0.29	0.26	0.16	0.29	0.16	0.3	0.19	0.27	0.19	0.23	0.14	0.28	0.2

TABLE C2. Performance metrics used in Fig. 4 for the model using YSU prior to the bias correction (WRF) and all the different regressors (as in Table C1). The different regressions are assigned a suffix: "avg" for regressions that were trained on the summertime-mean average of daily minimum, daily mean, or daily maximum temperatures and "tstep" for those that were trained with the temperatures at each daily time step.

	WRF	RF_{avg}	RF_{tstep}	LR_{avg}	LR_{tstep}	RD_{avg}	RD_{tstep}	LA_{avg}	LA_{tstep}	GB_{avg}	GB_{tstep}	DU_{avg}	DU_{tstep}
Mean													
RMSE	1.33	1.09	1.16	1.07	1.16	1.08	1.16	1.09	1.18	1.15	1.17	1.1	1.19
MAE	1.04	0.82	0.86	0.82	0.86	0.82	0.87	0.83	0.89	0.87	0.85	0.84	0.89
MB	−0.76	0	0.17	0	0.17	0	0.17	0.01	0.16	0.02	0.17	0.01	0.17
Pearson's r^2	0.09	0.07	0.07	0.07	0.07	0.08	0.07	0.08	0.07	0.05	0.07	0.09	0.07
Spearman's r	0.32	0.28	0.3	0.28	0.29	0.3	0.29	0.29	0.28	0.25	0.3	0.32	0.3
Min													
RMSE	1.58	1.05	1.06	1.04	1.06	1.05	1.07	1.06	1.09	1.12	1.09	1.06	1.09
MAE	1.27	0.83	0.83	0.81	0.82	0.82	0.83	0.82	0.84	0.88	0.84	0.83	0.84
MB	−1.17	0	−0.03	0	−0.03	0	−0.03	0	−0.03	0.04	−0.02	0	−0.03
Pearson's r^2	0.11	0.1	0.1	0.1	0.1	0.1	0.1	0.1	0.09	0.08	0.1	0.11	0.1
Spearman's r	0.37	0.35	0.37	0.35	0.36	0.34	0.35	0.34	0.34	0.31	0.36	0.36	0.35
Max													
RMSE	1.65	1.63	1.82	1.6	1.81	1.6	1.8	1.6	1.8	1.67	1.82	1.6	1.8
MAE	1.32	1.25	1.33	1.23	1.31	1.23	1.31	1.23	1.31	1.29	1.34	1.23	1.31
MB	−0.41	0	0.49	0	0.5	0	0.5	0.01	0.49	−0.01	0.49	0.01	0.5
Pearson's r^2	0.09	0.07	0.04	0.08	0.05	0.09	0.05	0.09	0.05	0.06	0.04	0.09	0.05
Spearman's r	0.32	0.27	0.23	0.29	0.24	0.31	0.25	0.3	0.26	0.25	0.22	0.31	0.26

Unauthenticated | Downloaded 06/05/24 10:06 AM UTC
